# Supplementary material for: Biofoundry-assisted expression and characterization of plant proteins
Source: Synth Biol (Oxf). 2021 Sep 11;6(1):ysab029. doi: 10.1093/synbio/ysab029 (PMC8529701; doi:10.1093/synbio/ysab029)
Supplement: ysab029_Supp [file ysab029_supp.zip › Supplementary Data - Figures S1-9 and Tables S1-S2.pdf]

Biofoundry-assisted expression and characterisation of plant proteins

Quentin M. Dudley[1], Yao-Min Cai[1], Kalyani Kallam[1], Hubert Debreyne[1], Jose A. Carrasco Lopez[1], Nicola J. Patron[1*]

[1] Engineering Biology, Earlham Institute, Norwich Research Park, Norwich, Norfolk, NR4 7UZ, UK

*nicola.patron@earlham.ac.uk

Supplementary Material

Supplementary Figures S1-9
Supplementary Tables S1-2

Supplementary Figure S1. Optimisation of tag cleavage by TEV protease (A) Comparison of cell-free expression levels for two plasmids encoding TEV protease (S219V). Protein concentration was measured by HiBiT; bar chart values represent averages (n=3) and error bars represent 1 standard deviation. (B) Protease cleavage of an MBP-sfGFP fusion protein at varying ratios of TEV protease (pEPQDKN0729) to target protein. The pEPQDKN0729 plasmid contains an N-terminal expression tag (similar to pEPQDKN0329) but has no C-terminal HiBiT tag. (C) Cell-free produced UGT73C5 without an N-terminal tag (pEPQDKN0437) or with an NET-SUMO N-terminal tag (pEPQDKN0440) was incubated for 16 hours with cell-free reaction expressing TEV protease (130 w/w, pEPQDKN0729), with cell-free reaction expressing sfGFP (130 w/w, pEPQDKN0248), or used directly (no overnight incubation) and production of geraniol glucoside assayed after 15 minutes incubation with at 30 °C. The 30 µL enzymatic reaction consisted of 4.5 pmol enzyme from ~13 µL of CFPS reaction, ~2 µL of CFPS reaction expression protease or sfGFP, along with 100 mM Tris-HCl (pH 8.0), 2 mM UDP-glucose, and 2 mM geraniol. Bar chart values represent averages (n=3) and error bars represent 1 standard deviation

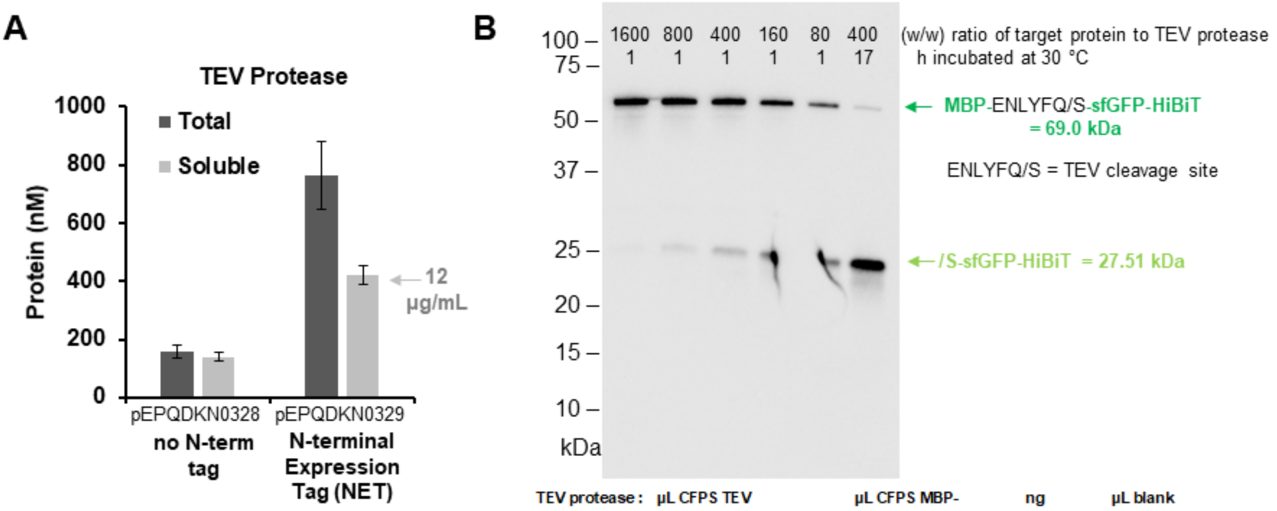

TEV protease : target protein (w/w)	µL CFPS TEV protease (12 µg/mL)	ng TEV protease	µL CFPS MBP-sfGFP (559 µg/mL) target protein	ng MBP-sfGFP target protein	µL blank CFPS rxn (no plasmid)
1:1600	0.14	1.7	5.00	2800	4.86
1:800	0.28	3.5	5.00	2800	4.72
1:400	0.57	6.9	5.00	2800	4.43
1:160	1.41	17.3	5.00	2800	3.59
1:80	2.83	34.6	5.00	2800	2.17

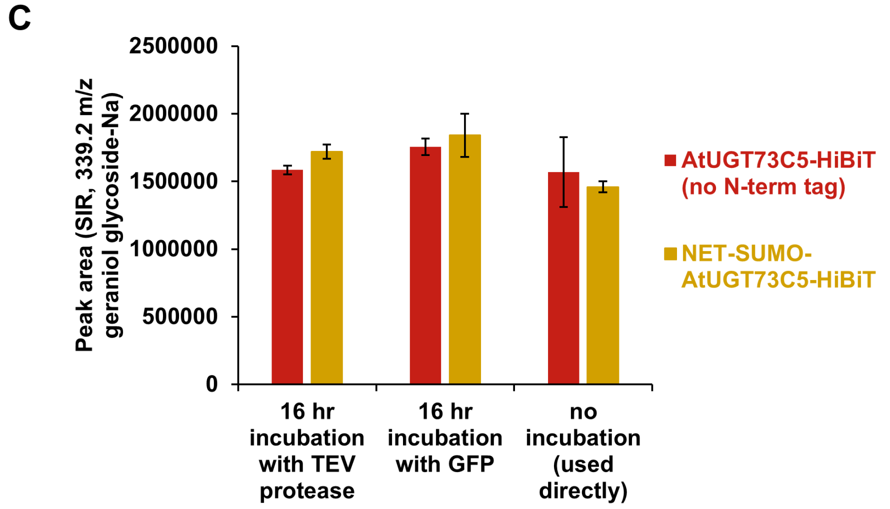

Supplementary Figure S2. Sequential improvement of Echo assembly protocol for consistent cell-free protein synthesis. All CFPS reactions are expressing pJL1-sfGFP (Addgene #69496) (A) The 2000 nL CFPS reaction consists of 265 nL of plasmid/water plus 1735 nL of a master mix containing the remaining reagents. The high viscosity of the master mix suggested that different sample plate type settings would transfer at different efficiency/consistency. A "droplet" test to transfer from the source plate to a foil-covered destination plate show that 384PP_AQ_GP2 and 384PP_DMSO2 were unable to transfer the master mix. (B) The sample plate type settings that successfully transferred master mix were compared in their ability to assemble CFPS reactions expressing sfGFP. 384PP_AQ_SP2 performed the most consistently, however, there is sufficient volume in the master mix source well to complete only 23 of 24 planned transfers. (C) Reducing the number of destination wells per source well avoided missed transfers, however, the long run time means the reactions assembled last (columns 23 and 24) have lower fluorescence. (D) To reduce the run time, we compared four different reaction volumes. 2000 nL is more consistent than 500 nL and produces the largest amount of protein. Pipetting master mix and *then* plasmid also minimized variability across the plate. (E) To further reduce the transfer time, the software program Echo Plate Reformat was used in place of Echo Cherry Pick to decrease the time taken for well scanning during master mix transfer.

A

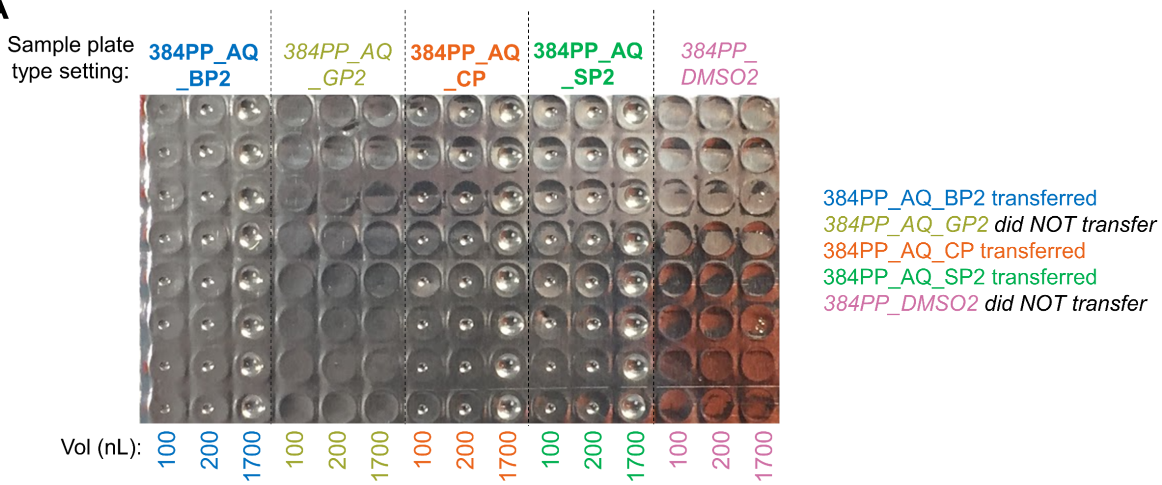

B

Sample plate type setting for CFPS master mix: variable (384PP_AQ_BP2, 384PP_AQ_SP2, 384PP_AQ_CP)

Destination wells per master mix source well: 24

Reaction volume: 2000 nL

Assembly order using Echo Cherry pick in single run: Plasmid/water first, CFPS master mix second (53 min total time)

384PP_AQ_BP2 (rows A–H, columns 1–12); 384PP_AQ_SP2 (rows A–H, columns 13–24); 384PP_AQ_CP (rows I–P, columns 1–24)

	1	2	3	4	5	6	7	8	9	10	11	12
A	58944	26314	27	59011	5238	98060	53000	19099	57640	58982	90492	30
B	55316	66896	22886	69035	19712	88292	73092	35139	47912	80668	76445	27
C	56904	64259	75021	65718	47222	100536	70750	36221	51758	63207	70950	27
D	55137	66250	83575	60767	72703	63987	64289	36704	64428	53378	99570	83104
E	49013	81757	27	65678	81399	85760	66395	90597	64533	63135	91258	30
F	68811	95904	28	12677	66187	65241	29034	31824	89956	90867	93061	28
G	23231	61685	30	18508	98853	28	20379	32405	90631	85559	98965	28
H	47974	62309	27	7541	39049	28	24080	70881	82486	96588	83511	27
I	59858	59722	44093	63568	61350	28	62666	64243	28	75606	59806	27
J	58054	60893	54127	60751	58973	27	64158	68425	27	58027	62222	27
K	56935	60465	56909	53879	60460	40218	54041	65023	38875	47158	59279	28
L	54523	64638	56225	53485	57673	63027	51620	64222	59825	54214	65125	27
M	55572	56098	26	58502	57251	29	55429	57542	27	58938	55830	28
N	51094	62681	26	63274	55724	28	57305	63845	28	69765	59932	27
O	54854	62367	27	57352	57352	27	55648	55061	27	59263	59878	27
P	56129	54429	27	60074	55215	27	59865	55815	27	62536	60532	27

	13	14	15	16	17	18	19	20	21	22	23	24
A	56594	58644	54596	51751	49543	46545	46303	40050	42215	39559	42149	39474
B	62917	61739	53473	66338	53451	52369	53323	56443	41405	45294	41536	39087
C	62708	58907	60203	58871	56325	51321	53800	50138	45984	43271	42100	40853
D	65230	58073	55994	58901	53598	55079	65994	56543	50706	51258	40368	49871
E	60571	59675	60219	58757	56614	58333	56086	54610	51636	50038	43343	40992
F	63219	66241	71226	72323	57835	58290	57135	61080	50809	52699	40889	41897
G	63092	58523	38522	61057	54463	48604	57022	54665	35364	50179	43926	36203
H	60016	56125	27	59612	59959	28	28255	61813	25	59566	44623	27
I	71968	63655	62493	53165	59628	27	67104	51898	52265	44026	49458	49795
J	59480	70343	62775	27	69642	27	70633	45385	49921	46665	51415	48854
K	56607	68796	59806	27	77096	50220	59063	43059	47898	48917	49791	52834
L	64219	56611	72819	28	72817	46747	45062	40701	47731	53035	51588	56662
M	64581	27	80571	49269	64893	53893	62814	54390	45953	51114	42328	54199
N	80616	28	69818	59768	58019	49051	57050	52904	58320	53079	53353	53649
O	64669	54981	63359	56863	56540	46907	26	53892	44736	47177	51985	48308
P	65441	58404	61752	53594	25	46183	27	46243	52070	44163	51389	51592

C

Sample plate type setting for CFPS master mix: 384PP_AQ_SP2

Destination wells per master mix source well: 20 or 21

Reaction volume: 2000 nL

Assembly order using Echo Cherry pick in single run: Plasmid/water first, CFPS master mix second (42 min total time)

	1	2	3	4	5	6	7	8	9	10	11	12	13	14	15	16	17	18	19	20	21	22	23	24
A	53148	71035	69352	72650	63443	66158	67962	69568	64508	66835	70181	67796	62197	65769	64202	55656	63907	67745	59772	60747	55808	58202	47639	38856
B	66367	66951	70940	70257	55516	67087	76722	69943	61704	69907	67937	70048	65702	69534	66471	52922	71516	67624	65427	65932	68514	59188	56142	42943
C	64807	68537	62777	69967	64454	68671	73318	69684	32850	69810	66317	66556	65138	66014	64069	62212	66162	67919	63600	65821	64162	65876	55865	40766
D	62110	67478	69819	71840	69278	70225	77662	74611	58605	71845	70700	65650	58968	69272	70201	61370	71426	65238	66571	53729	67318	64735	60420	42127
E	73794	67679	64835	68729												70074	65804	68164	65705	64352	57283	64751	58957	40774
F	66532	67874	70872	67884												72969	71415	67845	63494	69733	66304	66310	62676	41952
G	65590	59932	67371	69380												63455	65930	71565	63930	66506	57535	59468	59201	43697
H	69297	56439	69837	69875												70355	68489	68460	64608	70173	66350	66162	56149	40488
I	67949	62882	65510	66991												70964	59733	67445	62266	64055	38487	60664	58776	43969
J	70416	67342	68176	61509												71398	69322	67234	64660	63780	28	60277	57258	42404
K	68145	68691	65616	71264												69871	62651	63853	65787	66055	57070	61627	55276	39567
L	71040	68957	42025	66672												69077	71427	65652	62077	69002	60653	63186	57109	41212
M	64833	66619	68733	66968	65248	69298	69986	67633	65467	73627	64211	66288	65696	59895	59429	66454	64662	64738	64000	62456	62497	57034	55252	40039
N	70808	74139	64950	65589	70179	65992	57279	67061	64646	71565	63704	70005	58256	57921	68593	68000	69375	52604	65771	69929	61071	59364	50709	50307
O	63202	68582	65602	66640	66190	65464	61890	64387	63163	68573	61905	64853	59834	61290	60600	64372	63162	48448	58214	60603	63435	53352	54471	55055
P	68881	69742	61267	65938	67969	69173	68037	65813	47712	69699	55059	63753	61610	64374	67623	62660	64825	57244	57605	61807	51646	43921	44355	50387

Average	Standard deviation	std dev/ avg
63183	8526	13%

Lower fluorescence for wells with master mix added last →

D

Sample plate type setting for CFPS master mix: 384PP_AQ_SP2

Destination wells per master mix source well: variable

Reaction volume: 500, 1000, 1500, 2000 nL

Assembly order in sequence: CFPS master mix *first* using Echo Cherry Pick (19 min), plasmid/water *second* using Echo Cherry Pick (4 min)

2000 nL (rows A–H, columns 1–12)

Average	Standard deviation	std dev/ avg
61864	2874	5%

1000 nL (rows A–H, columns 13–24)

Average	Standard deviation	std dev/ avg
40111	1639	4%

	1	2	3	4	5	6	7	8	9	10	11	12	13	14	15	16	17	18	19	20	21	22	23	24
A	59269	61377	64036	61864	61664	63095	56817	62053	57794	58300	59934	63032	38008	39667	39520	39320	38371	36878	38486	39683	38986	39403	39122	38595
B	62763	59202	61434	59252	59609	60923	62356	64109	61219	60308	62926	61047	40258	38298	38885	39282	39699	37306	37767	40094	40062	39990	39564	41017
C	60983	61073	61657	58195	61355	64396	57828	61503	63300	67068	59778	62518	38023	39122	40149	39211	38738	38531	39776	40770	39701	39806	37941	38197
D	66479	56564	61725	54919	59240	57963	56915	59025	60694	61103	63711	61300	39404	36507	40264	40620	40219	39370	40581	39311	42714	41394	38187	40927
E	61710	61900	59201	61566	63058	65870	59799	61220	70214	64845	58014	63732	38926	41415	41344	40426	38651	40073	39150	39996	40170	39207	39018	38214
F	65896	61908	61881	61535	64345	63594	68346	61541	63579	61454	64478	61884	41003	40094	41803	40267	42374	42278	40388	41580	40673	40084	38564	43374
G	65795	54122	60917	62694	65671	65210	60331	62322	62443	63094	58795	58905	38523	42144	41916	42932	39721	44531	39355	40148	40633	41364	39764	38503
H	66003	60830	62377	61020	62277	60198	63385	63617	68424	64736	62114	68430	42384	38374	45143	43263	41692	41085	42349	44020	41126	41483	41529	41906
I	23162	23125	21043	24079	21677	22631	21494	22759	22324	21659	22224	22915	48670	53292	50768	52408	49426	53842	47390	46728	49397	51239	47764	56711
J	22618	21381	23820	22113	22253	22736	22764	22747	23245	22192	24112	23831	51695	53703	52159	50290	53025	53271	51479	50944	52439	51491	50760	60133
K	22871	22709	21357	24235	21669	23352	22313	21823	23200	22748	22109	22848	51341	48964	49144	53237	52945	51288	50716	51052	52613	52551	49266	51969
L	23504	21520	24600	20806	22090	21524	23180	22073	23398	21659	24274	22682	54267	53029	54718	53479	54530	53142	55671	52228	55599	52085	51620	53914
M	23546	23796	20619	22042	21275	20858	21629	20567	23235	22455	22230	20740	50880	52150	51082	51456	51149	52635	52809	48916	52563	50594	48959	46251
N	23236	21297	22764	21781	22001	21388	16282	20859	22591	21898	23750	22343	53377	52555	54305	55435	54680	53846	53800	52466	54386	51450	52738	51559
O	22437	21159	21711	21452	22281	6287	21075	20094	22194	20584	21528	21147	50015	48682	49915	51327	50270	50069	48840	45237	46819	51225	48931	48237
P	22711	21342	22568	21023	21253	20727	14343	20846	21508	20074	22695	22328	55569	49120	53406	49809	52401	48709	52283	52789	47016	50429	51741	53096

500 nL (rows I–P, columns 1–12)

Average	Standard deviation	std dev/ avg
21896	2143	10%

1500 nL (rows I–P, columns 13–24)

Average	Standard deviation	std dev/ avg
51608	2448	5%

E

Sample plate type setting for CFPS master mix: 384PP_AQ_SP2

Destination wells per master mix source well: 20 or 21

Reaction volume: 2000 nL

Assembly order in sequence : CFPS master mix *first* using Echo Plate Reformat (18 min), plasmid/water *second* using Echo Cherry Pick (7 min)

	1	2	3	4	5	6	7	8	9	10	11	12	13	14	15	16	17	18	19	20	21	22	23	24
A	58135	55662	59342	59409	61627	63875	62682	57120	60549	61440	63034	58848	62176	59200	66355	59891	61511	62395	60107	62666	58323	65433	61556	58198
B	33470	56097	62717	61844	59836	57013	61253	57835	60937	62867	60026	62173	57298	59496	59242	60749	59983	60565	58952	56642	64092	54669	57793	61010
C	50325	62450	60018	61107	61231	66045	63806	58925	64318	63461	64515	61832	63492	62629	62814	59497	62826	63281	62646	65083	64727	62700	65331	60873
D	62029	64641	62886	64686	61241	62277	64116	58924	61666	63914	63120	62634	61740	60227	62617	62390	61466	60022	60850	59314	63615	59123	58664	60273
E	50683	66872	61258	64703										65279	65132	59772	64289	70980	62529	59070	60998	61440	59570	61561
F	56935	60378	72331	65253										64554	69249	65554	66458	65152	62396	59330	66389	62201	59963	62701
G	55784	60281	58599	59576										64274	65918	61348	62566	62501	61117	61145	62541	60409	60386	67244
H	66777	59013	65048	63333										65246	62844	65618	57448	59154	56768	60745	63502	56233	60015	61803
I	61707	57451	57388	59761									4089	63963	64827	62546	57950	62585	53994	62146	62588	58626	59946	59586
J	61335	64195	68989	63497									1979	66603	59196	60155	51515	56044	55647	58189	67035	62137	58779	67838
K	61708	66750	60636	61253									2613	59854	57721	55448	55552	54196	57583	54947	59894	59659	57336	58232
L	57409	59766	68967	63133									68559	66455	56935	59975	54683	54102	54776	57210	65294	55824	52811	59090
M	60610	62489	57394	63004	60604	61265	61516	60245	58774	61756	52311	56373	62289	57827	56080	52152	56156	54941	56089	58819	60730	63885	60413	58176
N	51910	56106	64889	62087	61273	60388	59428	49879	52325	55963	54235	56334	55298	62727	54252	54673	52878	52961	54151	54183	54123	51184	55627	53903
O	52424	66070	59936	55169	61333	57547	58177	56266	53297	52949	55108	54741	57359	52147	55695	55036	53796	51679	54746	57135	54490	55177	55809	67795
P	54639	57226	61064	59816	57342	61469	58204	52512	55187	56880	52252	60497	58193	55586	45107	55505	48699	50592	54309	52497	55403	57609	55806	66977

Average	Standard deviation	std dev/ avg
59763	4484	8%

Supplementary Figure S3. Correlation of 2 µL vs 15 µL expression of various UDP-glycosyltransferases. (A-B) A subset of reactions from Figure 3A were assembled manually using handheld pipettes (15 µL reactions) or automated using an Echo 550 acoustic liquid handler (2 µL reactions). Total protein was measured by HiBiT. Values represent averages (n=3) and error bars represent 1 standard deviation.

A

		AtUGT73C5	NbUGT73A24	NbUGT73A25	NbUGT74T6	NbUGT74N4	NbUGT76A4	NbUGT85A73	NbUGT85A104	NbUGT94E7	NbUGT709J6	NbUGT71AT3
**Echo 2 µL**	no tag	427										27
	NET	251								75		
	S-tag-TEV	100			146	26		49			58	
	NET-SUMO-TEV	469	235	159			70		6			
**Manual 15 µL**	no tag	459										33
	NET	288								136		
	S-tag-TEV	176			289	39		106			155	
	NET-SUMO-TEV	473	222	212			212		12			

B

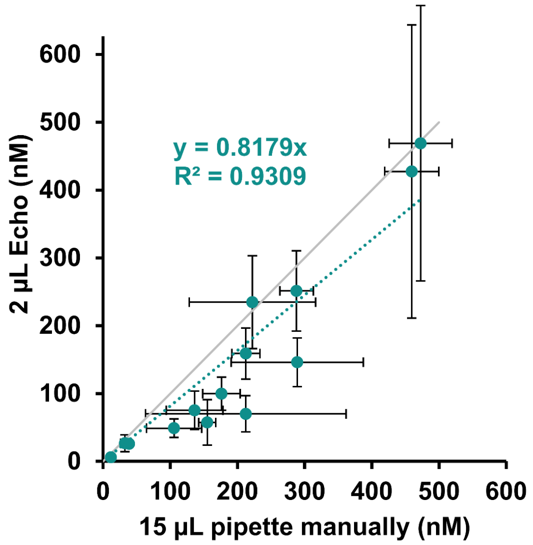

Supplementary Figure S4. Quantification of CcCPPase-HiBiT expression. Protein concentration was measured by HiBiT; bar chart values represent averages (n=3) and error bars represent 1 standard deviation.

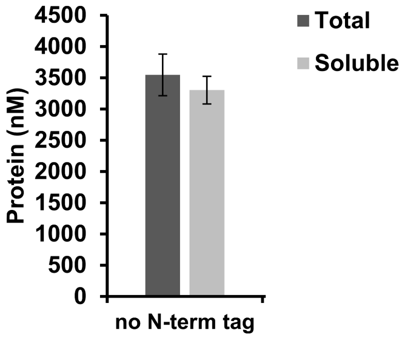

Supplementary Figure S5. Comparison of plasmid architecture for expressing sfGFP using the TNT® SP6 High-Yield Wheat Germ Protein Expression System L3260/L3261 (Promega) at 2 µL (A) and 15 µL (B) reaction volumes. The pEU expression plasmid contains a 331 fragment from the *E. coli* MG1655 genome which is positioned 3' of the gene of interest coding region. This genome fragment includes the terminator of the MG1655 folA and apaH coding regions. Using the "folA terminator" (architecture 1 or 2) produced more protein compared to using the T7 terminator (architecture 3). Values in panel A represent averages (n=4) and error bars represent 1 standard deviation.

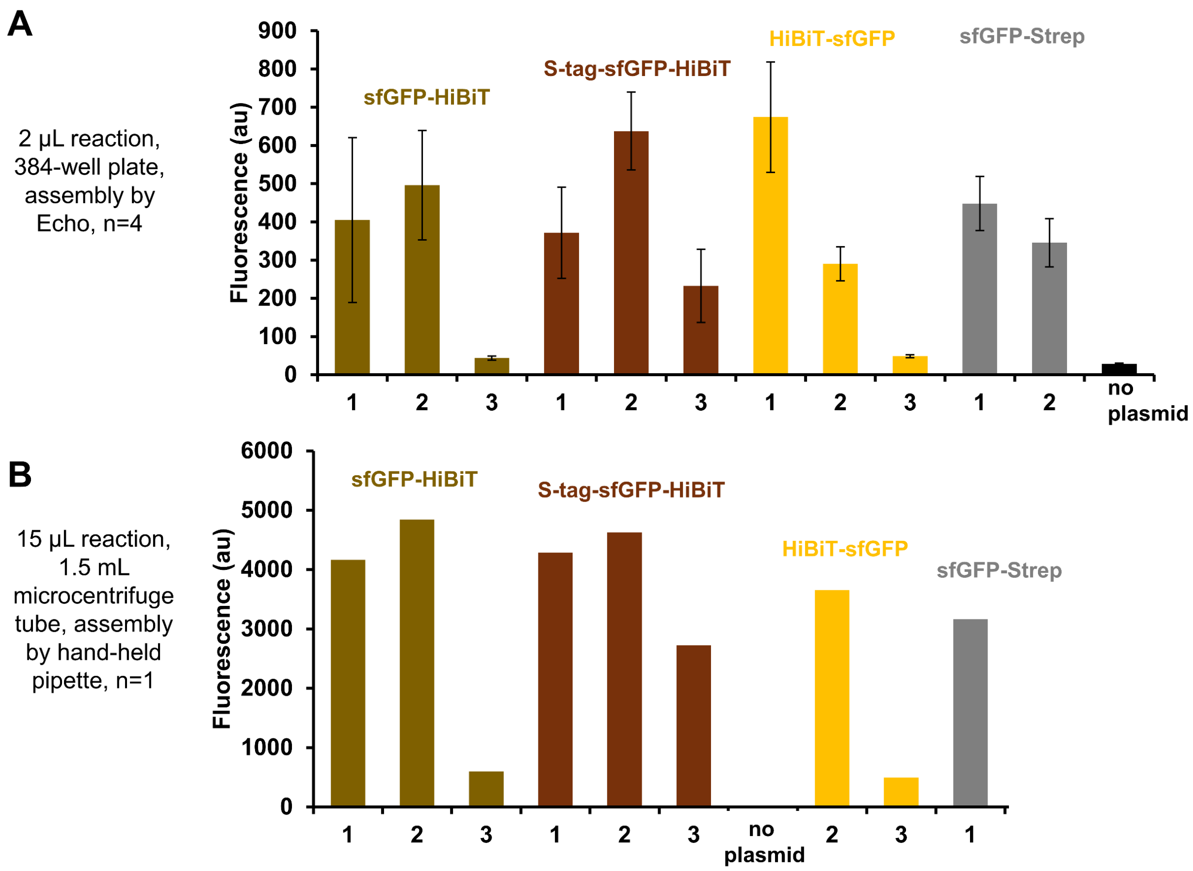

1: pEU-based, SP6 promoter, EO1 enhancer, folA terminator, carbR, pUC ori (pEPQD0CB0026, pEPQD0CB0246
2: pEU/pJL1 hybrid, SP6 promoter, EO1 enhancer, folA terminator, kanR, pUC ori (pEPQD0KN0284, pEPQD0KN0285)
3: pJL1-based, SP6 promoter, EO1 enhancer, T7 terminator, kanR, pUC ori (pEPQD0KN0282, pEPQD0KN0283)

Supplementary Figure S6. Measurement of geraniol glucoside over time. The reaction of purified AtUGT73C5 with 0.5 mM geraniol and 1 mM UDP-glucose is mostly complete after one hour.

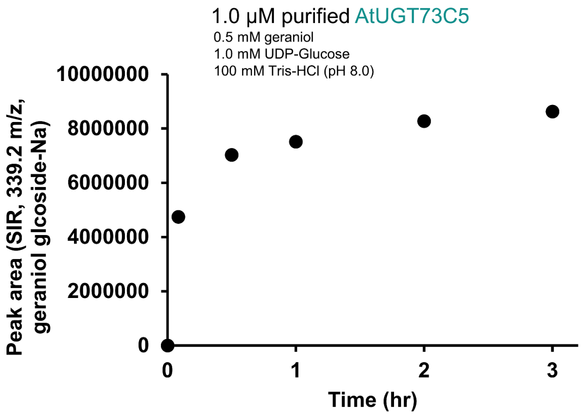

Supplementary Figure S7. Monitoring of six ions for geraniol-glucoside production. (A) The sodium (Na) adduct produces the strongest signal. (B) Geraniol-glucoside appears at a retention time of 5.87 minutes in the presence of AtUGT73C5

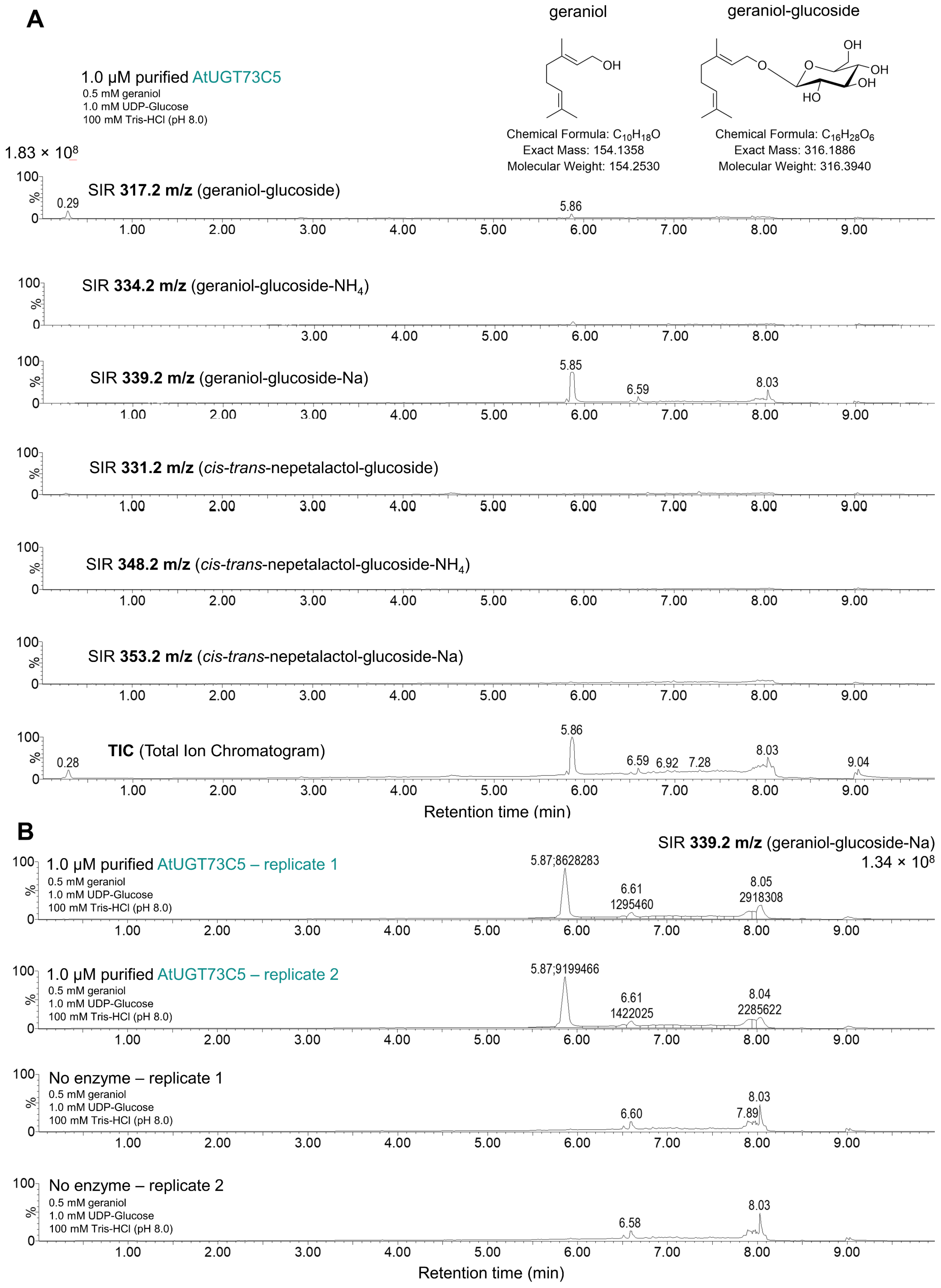

Supplementary Figure S8. Monitoring of six ions for *cis-trans*-nepetalatcol-glucoside production. (A) The sodium (Na) adduct producing the strongest signal. (B) *cis-trans*-nepetalctol-glucoside appears at a retention time of 5.15 minutes in the presence of AtUGT73C5

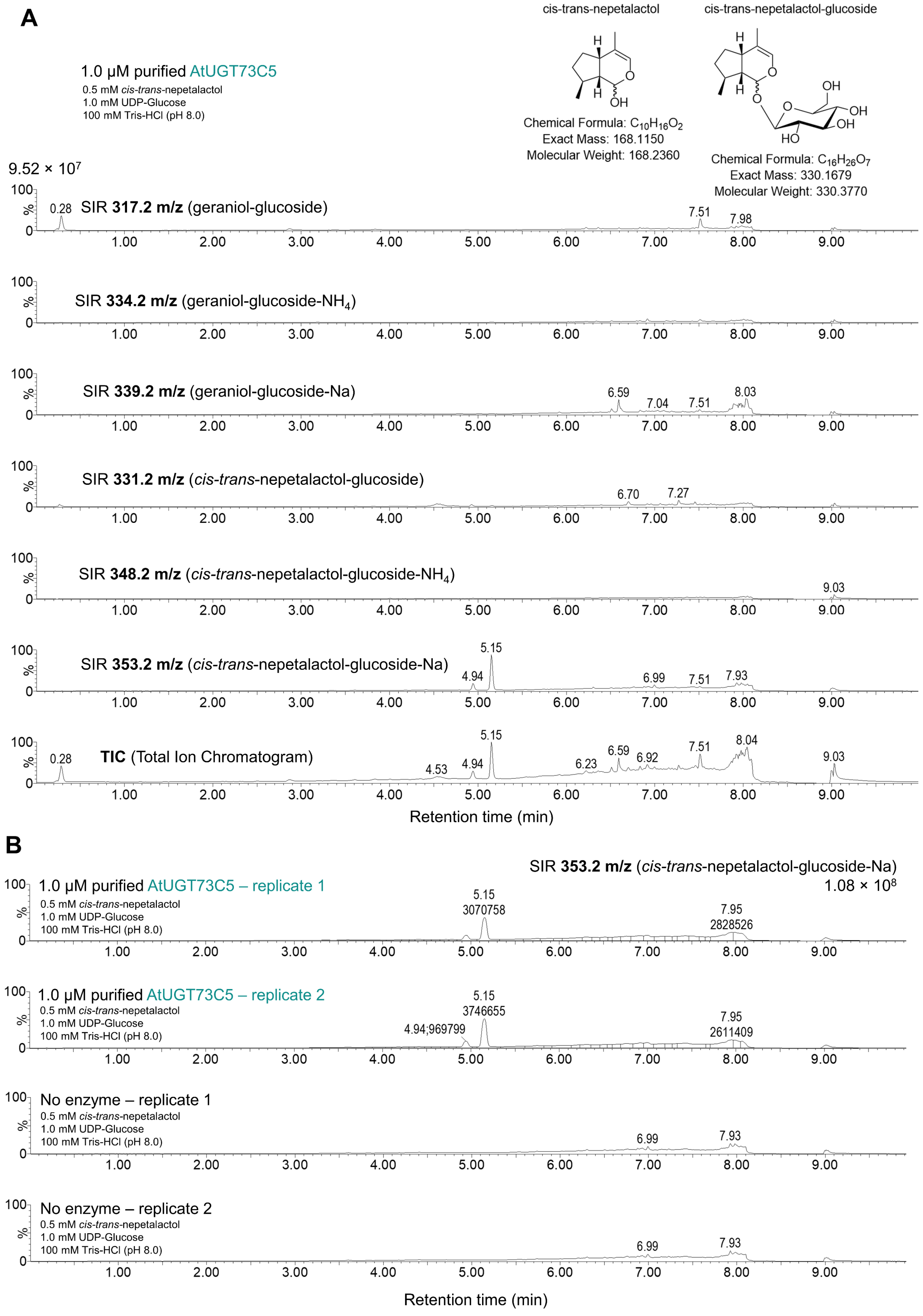

Supplementary Figure S9. GC-MS Total Ion Chromatogram of CcCPPase-HiBiT reaction with DMAPP. Ion scans of the TIC depicted in **Figure 4B** are compared to the NIST GC Method / Retention Index Library. (A) the peak at 8.08 minutes closely matches chrysanthemol B) the peak at 8.27 minutes closely matches lavandulol

A

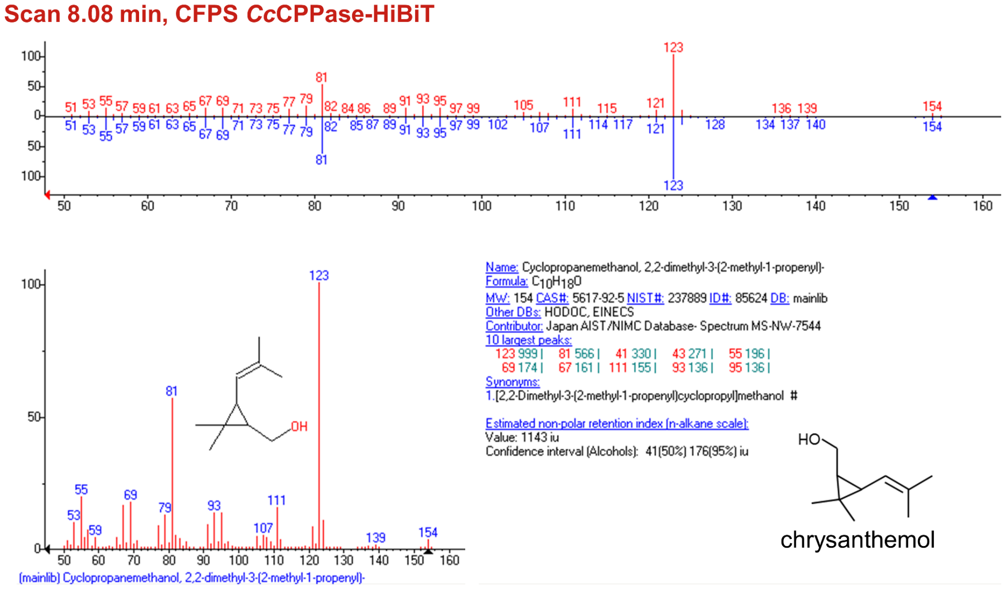

B

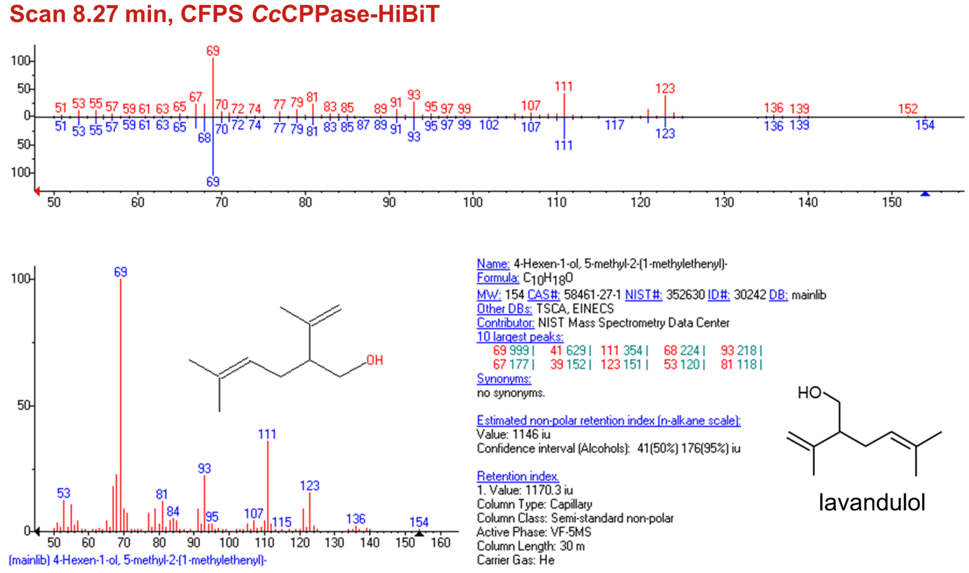

Supplementary Figure S10. Costs of cell-free protein synthesis reactions. (A) The reagents costs (when purchasing the smallest quantity available from the supplier) sufficient for 5,500 CFPS reactions (50 uL) using the *E. coli* PANOx-SP system. (B) Comparison of reagents costs for various cell-free reactions systems

A

Reagent	Price (£)	Mass (g)	Mass (g) required for 5500 50 µL reactions
Mg(Glu)2 (Sigma 49605)	£ 39.50	250	0.89
K(Glu) (Sigma G1149 BioReagent)	£ 37.50	100	7.53
ATP (Sigma A2383)	£ 44.30	1	0.19
GTP (Sigma G8877)	£ 136.00	0.25	0.13
UTP (Sigma U6625)	£ 194.50	0.5	0.13
CTP (Sigma C1506)	£ 147.50	0.25	0.13
Folinic acid (Sigma 47612 BioXtra)	£ 83.70	0.25	0.01
tRNA (Sigma TRNAMRE-RO ROCHE)	£ 148.00	0.1	0.05
PEP Roche 10108294001 via Sigma)	£ 450.00	2	1.96
NAD (Sigma N8535)	£ 129.00	0.75	0.06
CoA (Sigma C4282)	£ 266.00	0.1	0.06
Oxalic acid (Sigma P0963 BioXtra)	£ 23.32	50	0.10
Putrescine (Sigma P5780 (Cl salt) BioReagent)	£ 25.40	5	0.05
Spermidine (Sigma 85558 BioUltra)	£ 39.40	1	0.06
HEPES (Sigma H4034 BioPerformance)	£ 35.60	25	3.87
L-Valine (Sigma V0500)	£ 22.10	25	~1
L-Tryptophan (Sigma T0254)	£ 38.30	25	~1
L-Phenylalanine (Sigma P2126)	£ 48.60	100	~1
L-Isoleucine (Sigma I2752)	£ 48.60	25	~1
L-Leucine (Sigma L8000)	£ 22.90	25	~1
L-Cysteine (Sigma 30089 BioUltra)	£ 42.70	25	~1
L-Methionine (Sigma M9625)	£ 21.10	25	~1
L-Alanine (Sigma 05129 BioUltra)	£ 41.20	25	~1
L-Arginine (Sigma 11009 BioUltra)	£ 26.90	25	~1
L-Asparagine (Sigma A0884)	£ 22.30	25	~1
L-Aspartic acid (Sigma 11189 BioUltra)	£ 35.90	100	~1
L-Glutamic acid (Sigma 49449 BioUltra)	£ 30.80	100	~1
L-Glycine (Sigma G7126)	£ 23.40	100	~1
L-Glutamine (Sigma G3126 ReagentPlus 99% HPLC)	£ 51.30	100	~1
L-Histidine (Sigma H8000)	£ 27.50	25	~1
L-Lysine (Sigma L5626)	£ 20.50	100	~1
L-Proline (Sigma P0380)	£ 72.00	100	~1
L-Serine (Sigma 83949 BioUltra)	£ 69.10	25	~1
L-Threonine (Sigma T8625)	£ 35.70	25	~1
L-Tyrosine (Sigma T3754)	£ 22.50	50	~1
Magnesium acetate tetrahydrate (Sigma M5661)	£ 8.08	50	~1
Potassium acetate (Sigma P1190)	£ 36.10	100	~1
Media components for growing cells for lysate *	£ 192.00	-	-
**TOTAL:**	£ 2,759.30		
	£ 0.50	per 50 µL rxn	

* Media components include tryptone, yeast extract, sodium chloride, potassium phosphate, and dithiothreitol. The total cost is $3.25/mL lysate (see Silverman *et al Nat Rev Genet* 21 151-170 (2020)). Each reaction requires 300 mL/L, thus 5,500 50 µL reactions (275 mL) requires 268 USD = 192 GBP. Note that reagent costs for plasmid DNA have been excluded. All capital costs (dialysis, centrifugation, incubators) have been neglected.

B

Manufacturer	Source	Kit Description	Product #	# of 50 µL rxns	Cost	Expected yield (µg/mL)	Cost per 50 µL rxn
n/a	*E. coli*	S30 PANOx SP	-	5500	£ 2,759.30	100-1000	£ 0.50
Promega	*E. coli*	S30 T7 High-Yield Protein Expression System	L1115	24	£ 268.00	500	£ 11.17
Promega	Wheat Germ	TnT® SP6 High-Yield Wheat Germ Protein Exp. Sys.	L3260	40	£ 484.00	100	£ 12.10
NEB	*E. coli*	PURExpress® In Vitro Protein Synthesis Kit	E6800L	50	£ 2,059.00	10-200	£ 41.18

Supplemental Table S1. Description of cell-free expression plasmids assembled from acceptor plasmids and Level 0 DNA parts (phytobricks).

	Plasmid name	Description	Acceptor	N-term tag	CDS	C-term tag
**Figure 2A and 2C**: All plamids contain T7 promoter and T7 terminator needed for *E. coli* CFPS. **Figure 2B** uses CFPS reactions expressing pEPQDKN0313, pEPQDKN0310, pEPQDKN0314, pEPQDKN0316, pEPQDKN0318, and pEPQDKN0248 along with pEPQDKN0729 (TEV protease)	pJL1-sfGFP	Addgene #69496				
	pEPQDKN0248	sfGFP-HiBiT	pEPQD0KN0025	-	pEPQD0CM0296	pEPYC0CM0134
	pEPQDKN0308	GST-thrombin-sfGFP-HiBiT	pEPQD0KN0245	pEPQD0CM0541	pEPQD0CM0296	pEPYC0CM0134
	pEPQDKN0309	HiBiT-GST-thrombin-sfGFP	pEPQD0KN0245	pEPQD0CM0542	pEPQD0CM0296	pEPQD0CM0030
	pEPQDKN0310	GST-TEV-sfGFP-HiBiT	pEPQD0KN0245	pEPQD0CM0543	pEPQD0CM0296	pEPYC0CM0134
	pEPQDKN0311	MBP-polyargenine-Factor Xa-sfGFP-HiBiT	pEPQD0KN0245	pEPQD0CM0544	pEPQD0CM0296	pEPYC0CM0134
	pEPQDKN0312	HiBiT-MBP-polyargenine-Factor Xa-sfGFP	pEPQD0KN0245	pEPQD0CM0545	pEPQD0CM0296	pEPQD0CM0030
	pEPQDKN0313	MBP-TEV-sfGFP-HiBiT	pEPQD0KN0245	pEPQD0CM0546	pEPQD0CM0296	pEPYC0CM0134
	pEPQDKN0314	TrxA-TEV-sfGFP-HiBiT	pEPQD0KN0245	pEPQD0CM0547	pEPQD0CM0296	pEPYC0CM0134
	pEPQDKN0315	HiBiT-TrxA-TEV-sfGFP	pEPQD0KN0245	pEPQD0CM0548	pEPQD0CM0296	pEPQD0CM0030
	pEPQDKN0316	SUMO-TEV-sfGFP-HiBiT	pEPQD0KN0245	pEPQD0CM0549	pEPQD0CM0296	pEPYC0CM0134
	pEPQDKN0317	HiBiT-SUMO-TEV-sfGFP	pEPQD0KN0245	pEPQD0CM0550	pEPQD0CM0296	pEPQD0CM0030
	pEPQDKN0318	S-tag-TEV-sfGFP-HiBiT	pEPQD0KN0245	pEPQD0CM0281	pEPQD0CM0296	pEPYC0CM0134
	pEPQDKN0319	HiBiT-S-tag-TEV-sfGFP	pEPQD0KN0245	pEPQD0CM0551	pEPQD0CM0296	pEPQD0CM0030
	pEPQDKN0320	NET-GST-thrombin-sfGFP-HiBiT	pEPQD0KN0244	pEPQD0CM0541	pEPQD0CM0296	pEPYC0CM0134
	pEPQDKN0321	NET-GST-TEV-sfGFP-HiBiT	pEPQD0KN0244	pEPQD0CM0543	pEPQD0CM0296	pEPYC0CM0134
	pEPQDKN0322	NET-MBP-polyargenine-Factor Xa-sfGFP-HiBiT	pEPQD0KN0244	pEPQD0CM0544	pEPQD0CM0296	pEPYC0CM0134
	pEPQDKN0323	NET-MBP-TEV-sfGFP-HiBiT	pEPQD0KN0244	pEPQD0CM0546	pEPQD0CM0296	pEPYC0CM0134
	pEPQDKN0324	NET-TrxA-TEV-sfGFP-HiBiT	pEPQD0KN0244	pEPQD0CM0547	pEPQD0CM0296	pEPYC0CM0134
	pEPQDKN0325	NET-SUMO-TEV-sfGFP-HiBiT	pEPQD0KN0244	pEPQD0CM0549	pEPQD0CM0296	pEPYC0CM0134
	pEPQDKN0326	NET-S-tag-TEV-sfGFP-HiBiT	pEPQD0KN0244	pEPQD0CM0281	pEPQD0CM0296	pEPYC0CM0134
	pEPQDKN0327	NET-sfGFP-HiBiT	pEPQD0KN0024	-	pEPQD0CM0296	pEPYC0CM0134
	pEPQDKN0348	HiBiT-sfGFP	pEPQD0KN0245	pEPYC0CM0258	pEPQD0CM0296	pEPQD0CM0030
	pEPQDKN0729	NET-TEVprotease	pEPQD0KN0024	-	pEPQD0CM0539	pEPQD0CM0030
**Figure3A**: All plamids contain T7 promoter and T7 terminator needed for *E. coli* CFPS. All UGT coding sequenes were optimized for *E. coli* codon usage	pEPQDKN0437	AtUGT73C5-HiBiT	pEPQD0KN0025	-	pEPQD0CM0540	pEPYC0CM0134
	pEPQDKN0438	NET-AtUGT73C5-HiBiT	pEPQD0KN0024	-	pEPQD0CM0540	pEPYC0CM0134
	pEPQDKN0439	S-tag-TEV-AtUGT73C5-HiBiT	pEPQD0KN0245	pEPQD0CM0281	pEPQD0CM0540	pEPYC0CM0134
	pEPQDKN0440	NET-SUMO-TEV-AtUGT73C5-HiBiT	pEPQD0KN0244	pEPQD0CM0549	pEPQD0CM0540	pEPYC0CM0134
	pEPQDKN0441	TrxA-TEV-AtUGT73C5-HiBiT	pEPQD0KN0245	pEPQD0CM0547	pEPQD0CM0540	pEPYC0CM0134
	pEPQDKN0442	GST-thrombin-AtUGT73C5-HiBiT	pEPQD0KN0245	pEPQD0CM0541	pEPQD0CM0540	pEPYC0CM0134
	pEPQDKN0443	GST-TEV-AtUGT73C5-HiBiT	pEPQD0KN0245	pEPQD0CM0543	pEPQD0CM0540	pEPYC0CM0134
	pEPQDKN0444	MBP-TEV-AtUGT73C5-HiBiT	pEPQD0KN0245	pEPQD0CM0546	pEPQD0CM0540	pEPYC0CM0134
	pEPQDKN0453	NbUGT74T6-HiBiT	pEPQD0KN0025	-	pEPQD0CM0552	pEPYC0CM0134
	pEPQDKN0454	NET-NbUGT74T6-HiBiT	pEPQD0KN0024	-	pEPQD0CM0552	pEPYC0CM0134
	pEPQDKN0455	S-tag-TEV-NbUGT74T6-HiBiT	pEPQD0KN0245	pEPQD0CM0281	pEPQD0CM0552	pEPYC0CM0134
	pEPQDKN0456	NET-SUMO-TEV-NbUGT74T6-HiBiT	pEPQD0KN0244	pEPQD0CM0549	pEPQD0CM0552	pEPYC0CM0134
	pEPQDKN0457	TrxA-TEV-NbUGT74T6-HiBiT	pEPQD0KN0245	pEPQD0CM0547	pEPQD0CM0552	pEPYC0CM0134
	pEPQDKN0458	GST-thrombin-NbUGT74T6-HiBiT	pEPQD0KN0245	pEPQD0CM0541	pEPQD0CM0552	pEPYC0CM0134
	pEPQDKN0459	GST-TEV-NbUGT74T6-HiBiT	pEPQD0KN0245	pEPQD0CM0543	pEPQD0CM0552	pEPYC0CM0134
	pEPQDKN0460	MBP-TEV-NbUGT74T6-HiBiT	pEPQD0KN0245	pEPQD0CM0546	pEPQD0CM0552	pEPYC0CM0134
	pEPQDKN0461	NbUGT74N4-HiBiT	pEPQD0KN0025	-	pEPQD0CM0553	pEPYC0CM0134
	pEPQDKN0462	NET-NbUGT74N4-HiBiT	pEPQD0KN0024	-	pEPQD0CM0553	pEPYC0CM0134
	pEPQDKN0463	S-tag-TEV-NbUGT74N4-HiBiT	pEPQD0KN0245	pEPQD0CM0281	pEPQD0CM0553	pEPYC0CM0134
	pEPQDKN0464	NET-SUMO-TEV-NbUGT74N4-HiBiT	pEPQD0KN0244	pEPQD0CM0549	pEPQD0CM0553	pEPYC0CM0134
	pEPQDKN0465	TrxA-TEV-NbUGT74N4-HiBiT	pEPQD0KN0245	pEPQD0CM0547	pEPQD0CM0553	pEPYC0CM0134
	pEPQDKN0466	GST-thrombin-NbUGT74N4-HiBiT	pEPQD0KN0245	pEPQD0CM0541	pEPQD0CM0553	pEPYC0CM0134
	pEPQDKN0467	GST-TEV-NbUGT74N4-HiBiT	pEPQD0KN0245	pEPQD0CM0543	pEPQD0CM0553	pEPYC0CM0134
	pEPQDKN0468	MBP-TEV-NbUGT74N4-HiBiT	pEPQD0KN0245	pEPQD0CM0546	pEPQD0CM0553	pEPYC0CM0134
	pEPQDKN0469	NbUGT71AT3-HiBiT	pEPQD0KN0025	-	pEPQD0CM0554	pEPYC0CM0134
	pEPQDKN0470	NET-NbUGT71AT3-HiBiT	pEPQD0KN0024	-	pEPQD0CM0554	pEPYC0CM0134
	pEPQDKN0471	S-tag-TEV-NbUGT71AT3-HiBiT	pEPQD0KN0245	pEPQD0CM0281	pEPQD0CM0554	pEPYC0CM0134
	pEPQDKN0472	NET-SUMO-TEV-NbUGT71AT3-HiBiT	pEPQD0KN0244	pEPQD0CM0549	pEPQD0CM0554	pEPYC0CM0134
	pEPQDKN0473	TrxA-TEV-NbUGT71AT3-HiBiT	pEPQD0KN0245	pEPQD0CM0547	pEPQD0CM0554	pEPYC0CM0134
	pEPQDKN0474	GST-thrombin-NbUGT71AT3-HiBiT	pEPQD0KN0245	pEPQD0CM0541	pEPQD0CM0554	pEPYC0CM0134
	pEPQDKN0475	GST-TEV-NbUGT71AT3-HiBiT	pEPQD0KN0245	pEPQD0CM0543	pEPQD0CM0554	pEPYC0CM0134
	pEPQDKN0476	MBP-TEV-NbUGT71AT3-HiBiT	pEPQD0KN0245	pEPQD0CM0546	pEPQD0CM0554	pEPYC0CM0134
	pEPQDKN0477	NbUGT94E7-HiBiT	pEPQD0KN0025	-	pEPQD0CM0555	pEPYC0CM0134
	pEPQDKN0478	NET-NbUGT94E7-HiBiT	pEPQD0KN0024	-	pEPQD0CM0555	pEPYC0CM0134
	pEPQDKN0479	S-tag-TEV-NbUGT94E7-HiBiT	pEPQD0KN0245	pEPQD0CM0281	pEPQD0CM0555	pEPYC0CM0134
	pEPQDKN0480	NET-SUMO-TEV-NbUGT94E7-HiBiT	pEPQD0KN0244	pEPQD0CM0549	pEPQD0CM0555	pEPYC0CM0134
	pEPQDKN0481	TrxA-TEV-NbUGT94E7-HiBiT	pEPQD0KN0245	pEPQD0CM0547	pEPQD0CM0555	pEPYC0CM0134
	pEPQDKN0482	GST-thrombin-NbUGT94E7-HiBiT	pEPQD0KN0245	pEPQD0CM0541	pEPQD0CM0555	pEPYC0CM0134
	pEPQDKN0483	GST-TEV-NbUGT94E7-HiBiT	pEPQD0KN0245	pEPQD0CM0543	pEPQD0CM0555	pEPYC0CM0134
	pEPQDKN0484	MBP-TEV-NbUGT94E7-HiBiT	pEPQD0KN0245	pEPQD0CM0546	pEPQD0CM0555	pEPYC0CM0134
	pEPQDKN0485	NbUGT73A24-HiBiT	pEPQD0KN0025	-	pEPQD0CM0556	pEPYC0CM0134
	pEPQDKN0486	NET-NbUGT73A24-HiBiT	pEPQD0KN0024	-	pEPQD0CM0556	pEPYC0CM0134
	pEPQDKN0487	S-tag-TEV-NbUGT73A24-HiBiT	pEPQD0KN0245	pEPQD0CM0281	pEPQD0CM0556	pEPYC0CM0134
	pEPQDKN0488	NET-SUMO-TEV-NbUGT73A24-HiBiT	pEPQD0KN0244	pEPQD0CM0549	pEPQD0CM0556	pEPYC0CM0134
	pEPQDKN0489	TrxA-TEV-NbUGT73A24-HiBiT	pEPQD0KN0245	pEPQD0CM0547	pEPQD0CM0556	pEPYC0CM0134
	pEPQDKN0490	GST-thrombin-NbUGT73A24-HiBiT	pEPQD0KN0245	pEPQD0CM0541	pEPQD0CM0556	pEPYC0CM0134
	pEPQDKN0491	GST-TEV-NbUGT73A24-HiBiT	pEPQD0KN0245	pEPQD0CM0543	pEPQD0CM0556	pEPYC0CM0134
	pEPQDKN0492	MBP-TEV-NbUGT73A24-HiBiT	pEPQD0KN0245	pEPQD0CM0546	pEPQD0CM0556	pEPYC0CM0134
	pEPQDKN0493	NbUGT73A25-HiBiT	pEPQD0KN0025	-	pEPQD0CM0557	pEPYC0CM0134
	pEPQDKN0494	NET-NbUGT73A25-HiBiT	pEPQD0KN0024	-	pEPQD0CM0557	pEPYC0CM0134
	pEPQDKN0495	S-tag-TEV-NbUGT73A25-HiBiT	pEPQD0KN0245	pEPQD0CM0281	pEPQD0CM0557	pEPYC0CM0134
	pEPQDKN0496	NET-SUMO-TEV-NbUGT73A25-HiBiT	pEPQD0KN0244	pEPQD0CM0549	pEPQD0CM0557	pEPYC0CM0134
	pEPQDKN0497	TrxA-TEV-NbUGT73A25-HiBiT	pEPQD0KN0245	pEPQD0CM0547	pEPQD0CM0557	pEPYC0CM0134
	pEPQDKN0498	GST-thrombin-NbUGT73A25-HiBiT	pEPQD0KN0245	pEPQD0CM0541	pEPQD0CM0557	pEPYC0CM0134
	pEPQDKN0499	GST-TEV-NbUGT73A25-HiBiT	pEPQD0KN0245	pEPQD0CM0543	pEPQD0CM0557	pEPYC0CM0134
	pEPQDKN0500	MBP-TEV-NbUGT73A25-HiBiT	pEPQD0KN0245	pEPQD0CM0546	pEPQD0CM0557	pEPYC0CM0134

Supplemental Table S1 (continued)

	Plasmid name	Description	Acceptor	N-term tag	CDS	C-term tag
**Figure3A (continued)**: All plamids contain T7 promoter and T7 terminator needed for E. coli CFPS. All UGT coding sequenes were optimized for E. coli codon usage	pEPQDKN0501	NbUGT85A73-HiBiT	pEPQD0KN0025	-	pEPQD0CM0558	pEPYC0CM0134
	pEPQDKN0502	NET-NbUGT85A73-HiBiT	pEPQD0KN0024	-	pEPQD0CM0558	pEPYC0CM0134
	pEPQDKN0503	S-tag-TEV-NbUGT85A73-HiBiT	pEPQD0KN0245	pEPQD0CM0281	pEPQD0CM0558	pEPYC0CM0134
	pEPQDKN0504	NET-SUMO-TEV-NbUGT85A73-HiBiT	pEPQD0KN0244	pEPQD0CM0549	pEPQD0CM0558	pEPYC0CM0134
	pEPQDKN0505	TrxA-TEV-NbUGT85A73-HiBiT	pEPQD0KN0245	pEPQD0CM0547	pEPQD0CM0558	pEPYC0CM0134
	pEPQDKN0506	GST-thrombin-NbUGT85A73-HiBiT	pEPQD0KN0245	pEPQD0CM0541	pEPQD0CM0558	pEPYC0CM0134
	pEPQDKN0507	GST-TEV-NbUGT85A73-HiBiT	pEPQD0KN0245	pEPQD0CM0543	pEPQD0CM0558	pEPYC0CM0134
	pEPQDKN0508	MBP-TEV-NbUGT85A73-HiBiT	pEPQD0KN0245	pEPQD0CM0546	pEPQD0CM0558	pEPYC0CM0134
	pEPQDKN0509	NbUGT709J6-HiBiT	pEPQD0KN0025	-	pEPQD0CM0559	pEPYC0CM0134
	pEPQDKN0510	NET-NbUGT709J6-HiBiT	pEPQD0KN0024	-	pEPQD0CM0559	pEPYC0CM0134
	pEPQDKN0511	S-tag-TEV-NbUGT709J6-HiBiT	pEPQD0KN0245	pEPQD0CM0281	pEPQD0CM0559	pEPYC0CM0134
	pEPQDKN0512	NET-SUMO-TEV-NbUGT709J6-HiBiT	pEPQD0KN0244	pEPQD0CM0549	pEPQD0CM0559	pEPYC0CM0134
	pEPQDKN0513	TrxA-TEV-NbUGT709J6-HiBiT	pEPQD0KN0245	pEPQD0CM0547	pEPQD0CM0559	pEPYC0CM0134
	pEPQDKN0514	GST-thrombin-NbUGT709J6-HiBiT	pEPQD0KN0245	pEPQD0CM0541	pEPQD0CM0559	pEPYC0CM0134
	pEPQDKN0515	GST-TEV-NbUGT709J6-HiBiT	pEPQD0KN0245	pEPQD0CM0543	pEPQD0CM0559	pEPYC0CM0134
	pEPQDKN0516	MBP-TEV-NbUGT709J6-HiBiT	pEPQD0KN0245	pEPQD0CM0546	pEPQD0CM0559	pEPYC0CM0134
	pEPQDKN0517	NbUGT76A4-HiBiT	pEPQD0KN0025	-	pEPQD0CM0560	pEPYC0CM0134
	pEPQDKN0518	NET-NbUGT76A4-HiBiT	pEPQD0KN0024	-	pEPQD0CM0560	pEPYC0CM0134
	pEPQDKN0519	S-tag-TEV-NbUGT76A4-HiBiT	pEPQD0KN0245	pEPQD0CM0281	pEPQD0CM0560	pEPYC0CM0134
	pEPQDKN0520	NET-SUMO-TEV-NbUGT76A4-HiBiT	pEPQD0KN0244	pEPQD0CM0549	pEPQD0CM0560	pEPYC0CM0134
	pEPQDKN0521	TrxA-TEV-NbUGT76A4-HiBiT	pEPQD0KN0245	pEPQD0CM0547	pEPQD0CM0560	pEPYC0CM0134
	pEPQDKN0522	GST-thrombin-NbUGT76A4-HiBiT	pEPQD0KN0245	pEPQD0CM0541	pEPQD0CM0560	pEPYC0CM0134
	pEPQDKN0523	GST-TEV-NbUGT76A4-HiBiT	pEPQD0KN0245	pEPQD0CM0543	pEPQD0CM0560	pEPYC0CM0134
	pEPQDKN0524	MBP-TEV-NbUGT76A4-HiBiT	pEPQD0KN0245	pEPQD0CM0546	pEPQD0CM0560	pEPYC0CM0134
	pEPQDKN0525	NbUGT85A104-HiBiT	pEPQD0KN0025	-	pEPQD0CM0561	pEPYC0CM0134
	pEPQDKN0526	NET-NbUGT85A104-HiBiT	pEPQD0KN0024	-	pEPQD0CM0561	pEPYC0CM0134
	pEPQDKN0527	S-tag-TEV-NbUGT85A104-HiBiT	pEPQD0KN0245	pEPQD0CM0281	pEPQD0CM0561	pEPYC0CM0134
	pEPQDKN0528	NET-SUMO-TEV-NbUGT85A104-HiBiT	pEPQD0KN0244	pEPQD0CM0549	pEPQD0CM0561	pEPYC0CM0134
	pEPQDKN0529	TrxA-TEV-NbUGT85A104-HiBiT	pEPQD0KN0245	pEPQD0CM0547	pEPQD0CM0561	pEPYC0CM0134
	pEPQDKN0530	GST-thrombin-NbUGT85A104-HiBiT	pEPQD0KN0245	pEPQD0CM0541	pEPQD0CM0561	pEPYC0CM0134
	pEPQDKN0531	GST-TEV-NbUGT85A104-HiBiT	pEPQD0KN0245	pEPQD0CM0543	pEPQD0CM0561	pEPYC0CM0134
	pEPQDKN0532	MBP-TEV-NbUGT85A104-HiBiT	pEPQD0KN0245	pEPQD0CM0546	pEPQD0CM0561	pEPYC0CM0134
**Figure3B**: All plamids contain T7 promoter and T7 terminator needed for E. coli CFPS.	pEPQDKN0734	AtTGA1-HiBiT	pEPQD0KN0025	-	pEPYC0CM0470	pEPYC0CM0134
	pEPQDKN0735	NET-AtTGA1-HiBiT	pEPQD0KN0024	-	pEPYC0CM0470	pEPYC0CM0134
	pEPQDKN0736	S-tag-TEV-AtTGA1-HiBiT	pEPQD0KN0245	pEPQD0CM0281	pEPYC0CM0470	pEPYC0CM0134
	pEPQDKN0737	NET-6xHis-HRV3C-AtTGA1-HiBiT	pEPQD0KN0244	pEPMY0SP0002	pEPYC0CM0470	pEPYC0CM0134
	pEPQDKN0738	HiBiT-AtTGA1-strep	pEPQD0KN0245	pEPYC0CM0258	pEPYC0CM0470	pEPQD0CM0029
	pEPQDKN0739	AtTGA2-HiBiT	pEPQD0KN0025	-	pEPYC0CM0471	pEPYC0CM0134
	pEPQDKN0740	NET-AtTGA2-HiBiT	pEPQD0KN0024	-	pEPYC0CM0471	pEPYC0CM0134
	pEPQDKN0741	S-tag-TEV-AtTGA2-HiBiT	pEPQD0KN0245	pEPQD0CM0281	pEPYC0CM0471	pEPYC0CM0134
	pEPQDKN0742	NET-6xHis-HRV3C-AtTGA2-HiBiT	pEPQD0KN0244	pEPMY0SP0002	pEPYC0CM0471	pEPYC0CM0134
	pEPQDKN0743	HiBiT-AtTGA2-strep	pEPQD0KN0245	pEPYC0CM0258	pEPYC0CM0471	pEPQD0CM0029
	pEPQDKN0637	TaLUXA-HiBiT	pEPQD0KN0025	-	pSD0KN04	pEPYC0CM0134
	pEPQDKN0638	NET-TaLUXA-HiBiT	pEPQD0KN0024	-	pSD0KN04	pEPYC0CM0134
	pEPQDKN0639	S-tag-TEV-TaLUXA-HiBiT	pEPQD0KN0245	pEPQD0CM0281	pSD0KN04	pEPYC0CM0134
	pEPQDKN0640	NET-6xHis-HRV3C-TaLUXA-HiBiT	pEPQD0KN0244	pEPMY0SP0002	pSD0KN04	pEPYC0CM0134
	pEPQDKN0351	HiBiT-TaLUXA-strep	pEPQD0KN0245	pEPYC0CM0258	pSD0KN04	pEPQD0CM0029
	pEPQDKN0642	TaLUXB-HiBiT	pEPQD0KN0025	-	pSD0KN05	pEPYC0CM0134
	pEPQDKN0643	NET-TaLUXB-HiBiT	pEPQD0KN0024	-	pSD0KN05	pEPYC0CM0134
	pEPQDKN0644	S-tag-TEV-TaLUXB-HiBiT	pEPQD0KN0245	pEPQD0CM0281	pSD0KN05	pEPYC0CM0134
	pEPQDKN0645	NET-6xHis-HRV3C-TaLUXB-HiBiT	pEPQD0KN0244	pEPMY0SP0002	pSD0KN05	pEPYC0CM0134
	pEPQDKN0352	HiBiT-TaLUXB-strep	pEPQD0KN0245	pEPYC0CM0258	pSD0KN05	pEPQD0CM0029
	pEPQDKN0647	TaLUXD-HiBiT	pEPQD0KN0025	-	pSD0KN06	pEPYC0CM0134
	pEPQDKN0648	NET-TaLUXD-HiBiT	pEPQD0KN0024	-	pSD0KN06	pEPYC0CM0134
	pEPQDKN0649	S-tag-TEV-TaLUXD-HiBiT	pEPQD0KN0245	pEPQD0CM0281	pSD0KN06	pEPYC0CM0134
	pEPQDKN0650	NET-6xHis-HRV3C-TaLUXD-HiBiT	pEPQD0KN0244	pEPMY0SP0002	pSD0KN06	pEPYC0CM0134
	pEPQDKN0353	HiBiT-TaLUXD-strep	pEPQD0KN0245	pEPYC0CM0258	pSD0KN06	pEPQD0CM0029
**Figure 3C**	pEPQDKN0638	NET-TaLUXA-HiBiT, for *E. coli* CFPS	pEPQD0KN0024	-	pSD0KN04	pEPYC0CM0134
	pEPQDKN0641	TaLUXA-HiBiT, WG acceptor 2	pEPQD0KN0284	-	pSD0KN06	pEPYC0CM0134
	pEPQDKN0643	NET-TaLUXB-HiBiT, for *E. coli* CFPS	pEPQD0KN0024	-	pSD0KN05	pEPYC0CM0134
	pEPQDKN0646	TaLUXB-HiBiT, WG acceptor 2	pEPQD0KN0284	-	pSD0KN05	pEPYC0CM0134
	pEPQDKN0648	NET-TaLUXD-HiBiT, for *E. coli* CFPS	pEPQD0KN0024	-	pSD0KN06	pEPYC0CM0134
	pEPQDKN0651	TaLUXC-HiBiT, WG acceptor 2	pEPQD0KN0284	-	pSD0KN06	pEPYC0CM0134
	pEPQDKN0846	NET-TaLHYA-HiBiT, for *E. coli* CFPS	pEPQD0KN0024	-	pHR0CM03	pEPYC0CM0134
	pEPQDKN0848	TaLYHA-HiBiT, WG acceptor 2	pEPQD0KN0284	-	pHR0CM03	pEPYC0CM0134
	pEPQDKN0842	NET-TaNAMA1-HiBiT, for *E. coli* CFPS	pEPQD0KN0024	-	pHR0CM02	pEPYC0CM0134
	pEPQDKN0844	TaNAMA1-HiBiT, WG acceptor 2	pEPQD0KN0284	-	pHR0CM02	pEPYC0CM0134
**Figure 4A**	pEPQDCB0093	pOpin-His-AtUGT73C5-stop	pEPMY1CB0001	pEPMY0SP002	pEPQD0CM0265	pEPQD0CM0030
	pEPQDKN0248	sfGFP-HiBiT	pEPQD0KN0025	-	pEPQD0CM0296	pEPYC0CM0134
	pEPQDKN0437	AtUGT73C5-HiBiT	pEPQD0KN0025	-	pEPQD0CM0540	pEPYC0CM0134
	pEPQDKN0440	NET-SUMO-TEV-AtUGT73C5-HiBiT	pEPQD0KN0244	pEPQD0CM0549	pEPQD0CM0540	pEPYC0CM0134
	pEPQDKN0092	NET-AtUGT73C5-GFP	pEPQD0KN0024	-	pEPQD0CM0265	pEPQD0CM0027
**Figure 4B**	pEPKK1KN0203	CcCPPase-HiBiT	pEPQD0KN0025	-	pEPKK0CM0195	pEPYC0CM0134
**Figure 4C**	pEPQDKN0742	NET-6xHis-HRV3C-AtTGA2-HiBiT	pEPQD0KN0244	pEPMY0SP0002	pEPYC0CM0471	pEPYC0CM0134
**Supplementary Figure S1**	pEPQDKN0328	TEVprotease-HiBiT	pEPQD0KN0025	-	pEPQD0CM0539	pEPYC0CM0134
	pEPQDKN0329	NET-TEVprotease-HiBiT	pEPQD0KN0024	-	pEPQD0CM0539	pEPYC0CM0134
**Supplementary Figure S4:** All plasmids contain the SP6 promoter, an E01 translational enhancer, and a terminator suitable for the TNT SP6 High-Yield Wheat Germ Protein Expression System	pEPQDCB0334	sfGFP-HiBiT, WG acceptor 1	pEPQD0CB0026	-	pEPQD0CM0296	pEPYC0CM0134
	pEPQDKN0332	sfGFP-HiBiT, WG acceptor 2	pEPQD0KN0284	-	pEPQD0CM0296	pEPYC0CM0134
	pEPQDKN0330	sfGFP-HiBiT, WG acceptor 3	pEPQD0KN0282	-	pEPQD0CM0296	pEPYC0CM0134
	pEPQDCB0335	S-tag-sfGFP-HiBiT, WG acceptor 1	pEPQD0CB0246	pEPQD0CM0281	pEPQD0CM0296	pEPYC0CM0134
	pEPQDKN0333	S-tag-sfGFP-HiBiT, WG acceptor 2	pEPQD0KN0285	pEPQD0CM0281	pEPQD0CM0296	pEPYC0CM0134
	pEPQDKN0331	S-tag-sfGFP-HiBiT, WG acceptor 3	pEPQD0KN0283	pEPQD0CM0281	pEPQD0CM0296	pEPYC0CM0134
	pEPQDCB0752	HiBiT-sfGFP, WG acceptor 1	pEPQD0CB0246	pEPYC0CM0258	pEPQD0CM0296	pEPQD0CM0030
	pEPQDKN0350	HiBiT-sfGFP, WG acceptor 2	pEPQD0KN0285	pEPYC0CM0258	pEPQD0CM0296	pEPQD0CM0030
	pEPQDKN0349	HiBiT-sfGFP, WG acceptor 3	pEPQD0KN0283	pEPYC0CM0258	pEPQD0CM0296	pEPQD0CM0030
	pEPQDCB0055	sfGFP-strep, WG acceptor 1	pEPQD0CB0026	-	pEPQD0CM0296	pEPQD0CM0029
	pEPQDKN0759	sfGFP-strep, WG acceptor 2	pEPQD0KN0284	-	pEPQD0CM0296	pEPQD0CM0029

Supplemental Table S2. Nucleotide sequences of Level 0 DNA parts (phytobricks) encoding plant proteins

Plasmid name	Plasmid Description / Comments	DNA sequence of "insert" of the plasmid.<br>BsaI sites are coloured red.<br>Overhangs are coloured blue.
pEPQD0CM0540	CDS AtUGT73C5 (AT2G36800.1), codon optimised for *E. coli*	GGTCTCTAATGGTAAGCGAGACTACTAAGTCAAGCCCGTTGCATTTCGTCCT<br>GTTCCCGTTTATGGCCCAGGGTCATATGATCCCGATGGTCGACATTGCCCGT<br>CTGCTGGCACAACGCGGGGTAATTATTACCATCGTTACCACCCCGCATAACG<br>CGGCCCGCTTTAAGAACGTGCTGAATCGCGCGATCGAATCGGGTCTGCCGA<br>TTAATCTGGTACAGGTAAAATTCCCGTACTTGGAAGCGGGCCTGCAAGAGGG<br>CCAGGAAAACATTGACTCCCTGGATACCATGGAACGTATGATTCCGTTCTTCA<br>AGGCAGTGAATTTCTTAGAGGAGCCGGTGCAGAAACTTATCGAGGAAATGAA<br>TCCGCGCCCGAGTTGCTTAATCAGTGACTTCTGCCTGCCGTACACGTCGAAG<br>ATTGCGAAGAAATTTAACATTCCGAAAATTCTGTTTCACGGGATGGGCTGTTT<br>CTGCTTGCTCTGCATGCACGTGCTGCGTAAGAATCGCGAAATTCTCGATAAC<br>CTGAAAAGCGACAAAGAATTATTTACAGTACCAGACTTCCCGGACCGCGTGG<br>AGTTTACCCGCACTCAGGTACCAGTTGAGACTTACGTGCCTGCCGGGGATTG<br>GAAGGACATTTTCGACGGAATGGTGGAAGCCAACGAAACCAGTTACGGAGTC<br>ATTGTGAATAGTTTCCAGGAACTGGAACCGGCCTACGCGAAGGATTATAAAG<br>AAGTGCGTTCTGGAAAGGCTTGGACTATCGGGCCTGTAAGCCTGTGTAATAA<br>AGTTGGTGCGGATAAGGCCGAACGCGGTAATAAGTCTGATATCGACCAGGAC<br>GAATGTCTGAAGTGGCTGGACTCAAAGAAGCACGGAAGCGTTTTATATGTCT<br>GCCTGGGCAGCATTTGCAACTTACCACTGTCGCAGCTGAAAGAACTCGGGCT<br>GGGTCTTGAAGAGAGCCAGCGCCCATTTATCTGGGTGATTCGTGGCTGGGA<br>AAAGTATAAGGAACTGGTAGAATGGTTTAGCGAGTCGGGATTCGAGGACCGC<br>ATTCAGGACCGCGGCCTCTTGATTAAGGGCTGGAGCCCGCAGATGCTGATTC<br>TGAGCCACCCTTCGGTCGGTGGTTTTCTGACCCATTGCGGCTGGAATAGTAC<br>CCTCGAAGGTATTACGGCCGGCTTGCCTTTATTAACTTGGCCCTTATTTGCGG<br>ATCAGTTTTGTAACGAAAAGCTGGTTGTGGAAGTTTTAAAGGCGGGTGTTCG<br>CTCGGGCGTAGAGCAACCGATGAAGTGGGGCGAGGAAGAAAAGATCGGCGT<br>TTTAGTTGACAAGGAAGGTGTTAAGAAAGCGGTCGAGGAGCTCATGGGCGAA<br>TCAGACGACGCGAAGGAACGCCGCCGCCGTGCAAAGGAACTGGGCGACAG<br>CGCACATAAAGCCGTAGAGGAAGGTGGTAGCTCGCACTCAAATATTTCATTT<br>CTTCTGCAGGATATTATGGAGCTCGCCGAGCCGAACAACGGTTCGAGAGACC
pEPQD0CM0265	CDS AtUGT73C5 (AT2G36800.1), not codon optimised, contains a BbsI site	GGTCTCGAATGGTTTCCGAAACAACCAAATCTTCTCCACTTCACTTTGTTCTC<br>TTCCCTTTCATGGCTCAAGGCCACATGATTCCCATGGTTGATATTGCAAGGCT<br>CTTGGCTCAGCGTGGTGTGATCATAACAATTGTCACGACGCCTCACAATGCA<br>GCGAGGTTCAAGAATGTCCTAAACCGTGCCATTGAGTCTGGCTTGCCCATCA<br>ACTTAGTGCAAGTCAAGTTTCCATATCTAGAAGCTGGTTTGCAAGAAGGACAA<br>GAGAATATCGATTCTCTTGACACAATGGAGCGGATGATACCTTTCTTTAAAGC<br>GGTTAACTTTCTCGAAGAACCAGTCCAGAAGCTCATTGAAGAGATGAACCCT<br>CGACCAAGCTGTCTAATTTCTGATTTTTGTTTGCCTTATACAAGCAAAATCGCC<br>AAGAAGTTCAATATCCCAAAGATCCTCTTCCATGGCATGGGTTGCTTTTGTCT<br>TCTGTGTATGCATGTTTTACGCAAGAACCGTGAGATCTTGGACAATTTAAAGT<br>CAGATAAGGAGCTTTTCACTGTTCCTGATTTTCCTGATAGAGTTGAATTCACA<br>AGAACGCAAGTTCCGGTAGAAACATATGTTCCAGCTGGAGACTGGAAAGATA<br>TCTTTGATGGTATGGTAGAAGCGAATGAGACATCTTATGGTGTGATCGTCAAC<br>TCATTTCAAGAGCTCGAGCCTGCTTATGCCAAAGACTACAAGGAGGTAAGGT<br>CCGGTAAAGCATGGACCATTGGACCCGTTTCCTTGTGCAACAAGGTAGGAGC<br>CGACAAAGCAGAGAGGGGAAACAAATCAGACATTGATCAAGATGAGTGCCTT<br>AAATGGCTCGATTCTAAGAAACATGGCTCGGTGCTTTACGTTTGTCTTGGAAG<br>TATCTGTAATCTTCCTTTGTCTCAACTCAAGGAGCTGGGACTAGGCCTAGAGG<br>AATCCCAAAGACCTTTCATTTGGGTCATAAGAGGTTGGGAGAAGTACAAAGA<br>GTTAGTTGAGTGGTTCTCGGAAAGCGGCTTTGAAGATAGAATCCAAGATAGA<br>GGACTTCTCATCAAAGGATGGTCCCCTCAAATGCTTATCCTTTCACATCCATC<br>AGTTGGAGGGTTCCTAACACACTGTGGTTGGAACTCGACTCTTGAGGGGATA<br>ACTGCTGGTCTACCGCTACTTACATGGCCGCTATTCGCAGACCAATTCTGCA<br>ATGAGAAATTGGTCGTTGAGGTACTAAAAGCCGGTGTAAGATCCGGGGTTGA<br>ACAGCCTATGAAATGGGGAGAAGAGGAGAAAATAGGAGTGTTGGTGGATAAA<br>GAAGGAGTGAAGAAGGCAGTGGAAGAATTAATGGGTGAGAGTGATGATGCA<br>AAAGAGAGAAGAAGAAGAGCCAAAGAGCTTGGAGATTCAGCTCACAAGGCTG

		TGGAAGAAGGAGGCTCTTCTCATTCTAACATCTCTTTCTTGCTACAAGACATA<br>ATGGAACTGGCAGAACCCAATAATGGTTCGTGAGACC<br>**generated by PCR amplifying Arabidopsis thaliana Col1 cDNA using primers AGAGGTCTCTAATGGTTTCCGAAACAACCAA and AGAGGTCTCTCGAACCATTATTGGGTTCTGCCAGTTCC; the resulting PCR fragment was assembled into into pUPD2 (Addgene # 68161) using the restriction enzyme BsmBI* (Sarrion-Perdigones *et al. Plant Physiol.* 162, 1618-1634 (2013))
pEPQD0CM0552	CDS NbUGT74T6 (GenBank ID MT945322), codon optimised for *E. coli*	GGTCTCTAATGGATCTGCTCAACAATAAGAAATACGTGGCACACATCCTGGC<br>ACTGCCGTACCCTTCTCAGGGGCATATCAATCCGATGCTGCAGTTTTGTAAG<br>CGCCTGGTCAGCAAGTCAGTCAAAACGACCCTGGCGATCACCAATTTTATCA<br>GCCACTCTGTACGCCCCATCTCTATCAATGTGTCGATTGACACTATCTCGGAC<br>GGCTTTGATAAGGGCGGTTATGCCGAGGCAGATAGTATCGTTACTTACCTGG<br>AGCGTTTTAAGAAGATTGGGTCACAGACTCTGGAAGATCTGATTAAGAAGTAT<br>GAAAAGAGCGAATTCCCGATCACCTGCGTAATCTACGACGCGTTTATGCCGT<br>GGGCACTGGATGTTGCTAAGGACCACGGTCTGATCGGCTCATGCTTCTTTAC<br>CCAGGCGTGCTCCGTAAATTACATCTATTACTACGTCCACCATGGCAAGCTG<br>ACGCTGCCGATCTCCAGCCCGCCCGTACGTATCCCTGGCCTGCCTGAACTG<br>GAGTTACGTGACATGCCATCCTTTATCTACGTTCACGGCACCTACCCGGCCT<br>ACTTCGAACTGGTCCTTAACCAATTCATTAACGTTGAAAAGGCGGACTACGTG<br>TTCGTAAATAGCTTTTATAAATTAGAAGCAGAAGTGGTTGATGCCATGAGCAA<br>GGTGATCCCCATGTCAACGATTGGACCTACCCTGCCCAGTCTGTACCTGGAT<br>AACCGCGTAGAGAACGATACCGAGTACTGCTTATCTCTGTATCAGGTTGACG<br>CGAGTACGTGCATCAGTTGGCTTAACACGAAAACCGAGGGCAGCGTAGTGTA<br>CGTCGCATTCGGCAGCATGAGCACCATGGACAACGAGCAGATGGAAGAGAT<br>TGCGTGGGGCCTGAAGGCGACTAACTATTATTTCCTGTGGGTGGTCCGCACC<br>TGCGACGAGGCCAAGATCCCGAAGAACTTTATCGAAGAGACTAGCGAAAAGG<br>GGTTGGTTATTAACTGGAGCCCGCAGTTGCAGATTCTCAGTAACAAAGCCAT<br>CGGAATGTTCTTTAGCCACGGCGGCTGGAACTCGACCACCGAGGCGCTTTC<br>CTTGGGTGTACCCATGGTGGTTATGCCGTTATGGACTGACCAGACCACCAAC<br>GCCAAGCTGGTTCAGGACGTGTGGAGCGTGGGAGTTCGTGTCTCAGTGAAC<br>GAAAAGGGCTTCGCGGGGCGCGAGGAGATCGAAAAGTGCGTGCGCATTGTG<br>ATGGAAGGCGATAAAGGCAAGGAGATGAAGAAGAACGCTTTGAAGTGGAAA<br>GACCTGGCAAAAGAGGCAGTCAACGAGGGCGGTACGTCAGACAAGAACATC<br>GAGGAGTTCGTTTCGAAGTGCACCAAGGGTTCGAGAGACC
pEPQD0CM0553	CDS NbUGT74N4 (GenBank ID MT945323), codon optimised for *E. coli*	GGTCTCTAATGTCTACCACCCATAAGGCGCATTGTCTGATTTTACCGTACCCG<br>GTTCAGGGCCACATTAATCCGATGCTGCAGTTCAGCAAGCGCCTGGAGTCTA<br>AGGGAGTGAAGATTACCATTAGCCCAACCAAGTCATTTCTGAAAACAATGCAG<br>GAGCTGCCCACCTCTGTTTCGATTAAAGCGATCTCGGACGGTTATGATGACG<br>GCGGTATTGATCAGGCCGAGAGCTTTCTGGCATATATTACCCGTTTCAAGGA<br>AGTGGGGAGCGACACGCTCACACAGCTGATCAAGAAGCTGGAGTCATGCGA<br>ATATCCGGTTAACTGTATTGTATACGACCCGTTTCTCCCATGGGCAGTAGAGG<br>TCGCCAAAGACCTGGGCCTGGTGAACGCAGCCTTCTTTACTCAGAACTGCGT<br>GGTTGACAATATCTATTATCACGTGCACAAGGGCGTCTTAAAGCTGCCGCCG<br>ACGCAGGTAGACGGCCAGATTCTGATCCCGGGTTTGTCCTCAACCATCGAAA<br>GCAGCGACGTCCCGTCCTTCGAATCCAGCCCCCAGAGCGACAAGTTGGTCG<br>AGATGCTGGTCAACCAGTTTAGCAACTTAGAAAAGGTCGACTGGGTGTTGAT<br>TAATTCCTTTTACGAACTGGAAAAGGAAGTGATCGACTGGATGGCGAAATTCT<br>ACCCTATTAAAACGATCGGTCCTACCATTCCCTCGATGTATTTGGATAAACGC<br>CTGCCCAACGATAAGGAATACGGTTTGAGCTTATTTAAACCTATGGCGAAGG<br>AATGTCTCAACTGGCTGAACCCGCAGCCGATCTCCAGCGTTGTCTACGTGTC<br>GTTCGGGTCCATGGCGAAGCTGGAAGCGGAACAGATGGAAGAACTGGCGTG<br>GGGATCTAAGAACAGTAATAAGAATTTCCTGTGGGTTGTCCGTAGCACGGAA<br>GAGTCGAAGCTGCCTAAGAATTTCATTGAGGAGCTGAAGTCGGCCTCGGAAA<br>AGAAGGGCTTTGTAGTGAGCTGGTGCCCGCAGCTCCAGGTACTGGAGCACA<br>AGTCAATCGGCTGCTTCCTGACCCACAGCGGTTGGAACAGTACCCTTGAGGC<br>CATCAGCCTCGGCGTTCCTATTGTTACCATGCCGCAGTGGAGTGACCAGCCG<br>ACCAACGCCAAATTAGTTCAAGACGTCTGGGAAATGGGCGTGCGCGCGAAG<br>CAGGACGAGAAGGGGATCGTGCGCCGTGAGATCATCGAGGAGAACATTAAG<br>CTGGTCATGGAAGAGGAAAAGGGCAAGGTGATCCGCGAGAACGCCAAGAAG<br>TGGAAAGAGTTCGCCCGCAACGCCGTCGACGAGGGCGGCTCATCGGATAAG<br>AATATCGAGGAGTTTGTCTCAAGCCTGATCACGATCAGTGGTTCGAGAGACC
pEPQD0CM0554	CDS NbUGT71AT3 (GenBank ID MT945324),	GGTCTCTAATGAACGAATTAATCTTTATCCCTCTGGCGGGGCTCGGCCATCT<br>GGTGAGTGCAATTGAGTTCGCGAAGTTAGTGTTAAACCGCGACGACAAGGAC<br>CTGAGTATTAGCGTCTTAATTATGAAGCTCCCTCTGGATTACGGGGTACAGAA<br>CTTTATCCAATCCCTGAACAGCCAGCCGCGCCTGAAGTTTATTGACATTAGTC

	codon optimised for *E. coli*	TGGATGAAAAGACTAGCAGCACCTTTCTCAACAATCACGAGTCCTTTCTTTAC<br>GACTTTATTGACGGGCATAAGTCCAATGTTCGTGAGTACGTTCAGAACATCCC<br>ACGCCTGGCAGGATTTGTACTGGACATGTTCTGCACGTCAATGATCGATATT<br>GCGAATGAGTTCTCGGTGCCTTCTTACATCTACTTCGCGTCGAACGCTGCGT<br>TCCTGGGACTGTGCTTGCACTTCCAGGCCCTCACGAACGAACAGAACCTGGA<br>TACCTCAAAATATGTTAATACAGACAAAGAGCTCTCCATCCCCTACTTCAAGA<br>ACCCTTGCCCGACAAAGGTGCTGCCAAAACACCTGTTGAACAGCCGCCTGG<br>CGAGCACCCTGTTCTTCGACGGCATCCGCCGCTTCAAGGAAACCAAGGGCA<br>TCATCTTGAATACGTTCTTCGAACTGGAGAGTTTTAGCCTTCAAGCCCTGATG<br>GACTCGGAAATCGTGCCGACTATCTACCCCGTGGGCCCGGTCGTGTCTTTCG<br>CCAAGTCCGGCCACTTCCGCAACAACCCTTCTGAAACGGAGTCCATCATTAA<br>ATGGCTGGACGAACAGCCTGACCTTAGCGTTGTCTTTCTGTGTTTCGGATCTA<br>TGGGCAGCTTCGAAGCCGAACAGATCAAGGAGATTGCGACGGCCTTACAGC<br>ACTGTGGCCACCGCTTTCTGTGGAGCCTGCGCCGCAGCCCGCCCAAAGAGA<br>AGATTGACATTCCGTCTAACTATAATAACCTTGAGGAGATCCTGCCCAAGGAA<br>TTCCTGGAACGCACCAAGGGCATTGGCAAGGTTACGGGCTGGGCTCCGCAG<br>GTTGCGATCCTGAGTCACAAGTCTGTTGGCGGGTTCGTTTCGCACTGCGGCT<br>GGAACAGCATTCTGGAGAGCGTTTACTTCGGCGTACCCATTGCCACCTGGCC<br>GTTGTACGCGGAGCAACAGATGAACGCCTTCCTGCTGGTAAAGGAACTGGA<br>GATCGCGGAAGAGATCCGCATGGACTACTTCGTAGACTTCGTTGGACGCAAC<br>TCTAAGGTGGACATCGTGAGCGCCGAAGAGGTGGAAAGCGCCTTGCAGCGT<br>CTGATGGTTAAGAGCGAGGTCCGTGAAAAGGTAAAGAAGATGAAAGAAAAGG<br>CCCGTGTTGCCATGGAAGATGGCGGTTCGAGTTACCTGAGTCTGGCGTTACT<br>GATCAACGACATTATCAGTAACATCAGTGGTTCGAGAGACC
pEPQD0CM0555	CDS NbUGT94E7 (GenBank ID MT945325), codon optimised for *E. coli*	GGTCTCTAATGGACACCCAAGTCATCGAGTGTGGCAATAGCACGTCCCTGAA<br>AGTGTTGATGTTCCCCTGGCTTGCGTACGGCCACATTAGCCCGTTCCTGAAT<br>CTCGCGAAGAAGCTGGCCGATAAAGGCTTTCTCATCTATCTTTGCTCAACGC<br>CGATTAACCTGAAGTCGGTGGTGAAGAAGATTCCGGAGAAGTATTTCGTGTC<br>TATCCAATTAATCGAGTACCACTTGGCGGAGAGCCCGGAGTTACCGCCGCAC<br>TATCACACCACTAACGGTTTACCCCCACACTTGAACCATGCTTTACGTAAGGC<br>GCTCAAGCTGTCTAAGACCAATTTTAGTAAGATTCTCAAGTCCCTTAAGCCGG<br>ACCTGGTCGTGTACGACATCCTGCAACAGTGGGCCGAGGGATTGGCAAACG<br>AGCAGAATATCCCGGCGGTCAAACTGCTCACAAGCGGCGCGGCCGTGTTCT<br>CTTACTTCTTCAATCTTGTTAAGAAGCCAGAAGTAGAGTTCCCGTTCCCGGCA<br>ATCTACCACAAGAAGATCGAACTCGTGAAGCTTGGGGAGATGATGGCTAAGA<br>GTGCGAAGGAGAAGGAGAGCGACGACGTCGGTCCCTTCACCGAGGGCAACA<br>TGCAGATTATGCTGATGTCCACAAGTCGCATCATCGAAGCCAAGTATATTGAC<br>TACAGCTCAGAGCTGAGTAACTGGAAGGTCATCCCGGTGGGGCCGCCTGTG<br>CAGGACTCGATGGCAAACGGTACCGACGATGTAGAACTGTTCGACTGGCTG<br>GGCAAGAAGGACGAAAACTCTACAGTGTTCGTTAGCTTCGGTAGCGAATACT<br>TCCTGACCAAGGAAGATCGTGAGGAGATTGCATTCGGTCTGGAATTCAGCAA<br>CGTCAACTTTATTTGGGTAGTGCGTTTCCCCAACGGAGAGGAGCAGAACCTG<br>GAGAACGCCCTGCCCCAGGGGTTCCTGGAGCGTATCGGTGAGCGTGGGCG<br>CGTACTGAATAAGTGGGCACCGCAACCGCGTATCTTAAACAATCCAAACATT<br>GGCGGTTTCATTTCGCACTGCGGCTGGAACTCGGTCATGGAGTCCGTCGACT<br>TCGGCGTGCCGATTATTGCCATGCCGATGCACCTCGACCAGCCCGTTAACGC<br>CCGCCTCATGGCCGAGCTGGGTGTGGCAGTCGAAATCGTCCGCGGTGATGA<br>CGGTAAAATCCACCGCGAGGAAATCGCCCAGGTGCTGGAGAACGTAATTGC<br>CGGTAAGATTGGTGAGAACCTGCGTACGAAGGTGAAGGACGTATCGAAGAA<br>GCTTAAGAGTGTTCGCGCCGAGGAAACCGACGTGGTGGCAGAGGAGCTGAT<br>CCATTTCTGCAAAACATCCAACAAATGCAAGGGTTCGAGAGACC
pEPQD0CM0556	CDS NbUGT73A24 (GenBank ID MT945326), codon optimised for *E. coli*	GGTCTCTAATGGGGCAACTGCACATCTTCTTCTTTCCCATGATGGCCCACGG<br>ACATATGATCCCGACCTTAGATATGGCAAAACTTTTCGCATCCCGCGGCGTG<br>AAGGCAACCATTATTACCACCCCGCTGAACGAGAGTGTGTTTAGCAAGGCCA<br>TCCAGCGCAATAAACACCTCGGCATTGAGATTGAGATCCGCCTGATTAAGTTT<br>CCGGCAGTGGAGAATGATCTGCCGGAAGAGTGTGAGCGTCTGGACCAGATT<br>CCGAGCGACGAAAAGCTGCCTAACTTCTTCAAGGCGGTCGCTATGATGCAGG<br>AGCCGCTGGAAAAGTTAATCCAGGAGTGTCGTCCGAACTGCCTCGTGTCAGA<br>CATGTTTCTGCCATGGACGACCGACAGCGCTGCGAAGTTCAATATTCCTCGT<br>ATTGTGTTTCACGGAACGTCCTTCTTCGCCCTGTGTGTCGAAAACTCGGTGC<br>GTTTAAACAAACCGTTTAAGAACGTTTCGAGCGACAGCGAGACATTCGTGGT<br>GCCCAACCTTCCGCATGAGATCAAGCTTACTCGTACTCAAGTTAGTCCCTTTG<br>AACGCTCAGGCGAGGAAACGGCAATGACTCGCATGATTAAGACCGTGCGCG<br>AGAGTGACTCGAAAAGTTACGGTGTGGTCTTTAATTCATTTTACGAACTGGAG<br>ACTGACTACGTGGAGCACTACACGAAAGTCTTGGGCCGTCGCGCCTGGGCC

		ATCGGTCCACTTTCTATGTGTAATCGTGATATCGTGGACAAGGCGGAGCGCG<br>GTAAGAAGTCTAGCATCGACAAGCATGAATGTCTTAAGTGGCTGGACAGCAA<br>GAAGCCATCCAGTGTGGTCTATATCTGCTTTGGTTCAGTCGCCAACTTTACGG<br>CCAGCCAGTTGCATGAGCTGGCCATGGGCATCGAGGCCTCTGGCCAGGAGT<br>TTATCTGGGTGGTGCGTACCGAGCTTGATAATGAGGACTGGCTGCCGGAAG<br>GGTTTGAGGAGCGTACCAAGGAAAAGGGGCTGATTATTCGCGGCTGGGCGC<br>CGCAGGTGCTTATCCTCGACCATGAGTCCGTCGGCGCGTTCGTAACTCACTG<br>CGGTTGGAACTCTACGCTCGAGGGTGTGAGCGGTGGGGTGCCGATGGTTAC<br>CTGGCCGGTGTTCGCAGAACAGTTCTTTAACGAGAAACTGGTAACCGTAGTG<br>TTAAAGACGGGTGCGGGAGTGGGCTCAATCCAATGGAAACGTAGTGCATCC<br>GAGGGTGTTAAGCGTGAGGCGATTGCAAAAGCCGTTAAACGTGTCATGGTTT<br>CGGAAGAGGCTGACGGGTTTCGCAATCGCGCAAAGGCCTATAAAGAGATGG<br>CGACCAAGGCGATCGAGAAGGGCGGTTCATCGTATACCGGCCTCACAACAC<br>TGCTCGAGGACATCAGCACTTACTCATCGACGGACCACGGTTCGAGAGACC
pEPQD0CM0557	CDS NbUGT73A25 (GenBank ID MT945327), codon optimised for *E. coli*	GGTCTCTAATGGGTCAACTCCATTTCTTCTTCTTCCCGATGATGGCGCAAGG<br>GCATATGATTCCAACGCTGGATATGGCCAAATTGGTGGCATCTCGTGGCGTC<br>AAAGCCACAATTATCACCACCCCATTGAACGAATCAGTGTTTTCGAAAAGCAT<br>CCAGCGTAACAAACATTTGGGGATCGAAATTGAAATTCGCTTGATTAAATTTC<br>CTGCAATTGAGAACGACCTGCCGGAAGAATGCGAGCGTTTGGATCAAATTCC<br>GTCTGACGAGAAATTACCTAATTTCTTCAAAGCCACGGCAATGATGCAGGAAC<br>CGCTGGAACAGCTGATTGAAGAATGCCGTCCAAATTGCCTCGTATCCGACAT<br>GTTCTTGCCTTGGACCACGGACAGTGCAGCCAAATTTAACATTCCGCGTATT<br>GTGTTCCACGGCACTTCGTTCTTTGCTCTCTGTGTTGAAAACAGCGTGCGTCT<br>GAATAAACCCTTTAAGAATGTGTCCAGCGATAGCGAAACCTTTGTTGTACCGA<br>ATTTGCCTCATGAGATTAAACTCACGCGCACGCAAGTTTCTCCGTTTGAACAG<br>AGCGGTGAAGAAACGACTATGGCACGCATGATCAAGACAGTATGGGAATCCG<br>ATTCTCGCTCTTACGGGGTGGTGTTTAACTCATTCTACGAGCTGGAAACGGAT<br>TACGTAGAACATTACACCAAGGTTTTAGGCCGTCGCGCCTGGGCCATTGGCC<br>CTCTTTCGATGTGCAATCGCGACATTGAAGATAAAGCAGAACGCGGGAAGAA<br>ATCAAGCATTGACAAGCACGAATGCTTGAAATGGCTGGATTCGAAGAAACCG<br>TCTAGCGTCGTGTATATTTGCTTCGGCAGCGTGGCAAACTTTACTGCAAGTCA<br>GTTGCATGAACTGGCAATGGGCATTGAAGCGTCGGGCCAAGAATTCATCTGG<br>GTTGTGCGTACTGAGTTGGATAACGAAGATTGGCTGCCCGAAGGCTTCGAGG<br>AGCGTACTCGTGAGAAAGGCCTTATTATCCGCGGGTGGGCCCCGCAGGTGG<br>TGATTTTAGATCACGAATCAGTGGGCGCGTTCGTCACTCATTGCGGATGGAA<br>CTCCACGTTAGAGGGCGTTAGCGGTGGTGTACCTATGGTTACCTGGCCGGTA<br>TTCGCTGAGCAATTCTTCAATGAAAAGTTAGTTACCGAAGTGCTGCGTACCGG<br>TGCGGATGTGGGCTCTATTCAATGGAAACGCTCAGCGTCAGAAGGCGTCAAA<br>CGCGAAGCGATCGCCAAAGCCATTCAGCGTGTCATGGTATCGGAAGAGGCA<br>GAGGGCTTCCGTAACCGTGCAAAAGCATATAAAGAAATGGCTCGCAAAGCAG<br>TAGAAGAGGGCGGCAGCAGTTACACCGGCCTGACCACGCTGCTGGAAGATA<br>TCAGCACCTACAGTAGCACCGGTCATGGTTCGAGAGACC
pEPQD0CM0558	CDS NbUGT85A73 (GenBank ID MT945328), codon optimised for *E. coli*	GGTCTCTAATGGGATCGATTGGCGCAGAACTTACAAAACCTCATGCAGTGTG<br>CATTCCGTACCCGGCACAAGGTCATATCAACCCGATGTTAAAATTAGCCAAAA<br>TTCTGCACCACAAAGGCTTCCATATTACCTTTGTCAACACGGAATTTAACCAT<br>CGTCGCCTTCTGAAGTCCCGCGGCCCGGATAGCTTGAAGGGGCTGAGTAGC<br>TTTCGTTTCGAAACCATTCGTGACGGCCTGCCACCCTGTGAAGCAGATGCCA<br>CGCAGGACATCCCCTCGCTGTGTGAATCGACTACCACAACGTGCCTGGGCC<br>CATTTAAAGATCTGTTAGCCAAATTGAATGATACCAATACTAGCAATGTTCCCC<br>CGGTGAGCTGTATTGTGAGCGACGGCGTGATGTCATTTACACTGGCCGCAG<br>CACAAGAGTTAGGCGTGCCAGAAGTACTGTTTTGGACCACTTCGGCGTGTGG<br>ACTGTTAGGATACATGCACTACTACAAAGTGATTGAAAAGGGCTATGCTCCTC<br>TGAAAGATGCTACGGACTTGACCAATGGATACCTGGAGACTACCCTGGATTT<br>TATTCCAGGAATGAAAGATGTCCGTCTGCGCGATCTTCCGAGCTTTCTGCGT<br>ACCACCAATCCTGATGAGTTTATGATCAAATTTGTTCTCCAGGAAACCCAACG<br>CGCTCGCAAGGCCAGTGCCATCATTCTTAACACGTTCGAAACCTTAGAAGCC<br>GAGGTGCTGGAAACTCTTCGCAATTTGTTACCACCTGTGTATCCTATCGGCC<br>CGCTGCATTTCCTGGTTAAGCATGCAGACGATGAGAACCTGAAGGGTTTACG<br>TAGCAGTCTTTGGAAAGAAGAACCCGAATGTATCCAGTGGCTTGACACAAAG<br>GAACCTAACTCGGTTGTCTACGTGAATTTCGGGAGCATCACTGTTATGACTCC<br>GAACCAGTTAATTGAATTTGCTTGGGGCTTGGCAAACTCCCAGCAGACCTTC<br>CTGTGGATTATTCGTCCGGATATTGTTTCGGGTGATGCCAGCATCTTGCCGC<br>CAGAATTTGTGGAAGAAACCAAGAATCGTGGAATGCTGGCGTCATGGTGTAG<br>CCAGGAAGAAGTGTTGTCGCACCCGGCAATTGGCGGTTTTCTGACGCACAG<br>CGGCTGGAACAGCACCTTGGAATCTATTTCGAGCGGTGTACCGATGATTTGT<br>TGGCCCTTCTTTGCTGAACAACAAACCAATTGTTGGTTTAGCGTGACTAAGTG<br>GGATATCGGAATGGAAATTGACAGTGATGTTAAACGTGATGAGGTTGAGTCT

		CTTGTACGTGAGCTTATGGTTGGCGAGAAAGGCATTAAAATGAAGAAGAAGG<br>CTATGGAGTGGAAGGAACTGGCGGAAGAGAGCGCGAAAGAACACTCCGGAC<br>TTTCCTATGTCAATATTGAAAAGGTTGTGAACGATATTCTGCTGAGCAGCAAA<br>CATGGTTCGAGAGACC
pEPQD0CM0559	CDS NbUGT709J6 (GenBank ID MT945329), codon optimised for *E. coli*	GGTCTCTAATGCTCAGCATGGACAGCAGCGCATTTGTTCCGCACGTGGCAAT<br>TTTCCCGTTTCCGGCGCAAGGACACGTTAATAGCATGCTGAAGCTGGCGCAA<br>CTCCTGTCGGTGTCTAACTTCCATGTGAGCTTCCTGGTGACGGTGGATACGC<br>ACGATCGTCTGTTAAATCATACTGACGTGCTTTCTCGCTTCGGCAGTGAGTTT<br>CATCTGCAAGAGCTGCCCGTGGGCATCAGCTTGGACGAAATGAACACCCGC<br>GACGGCGTGGCGAAACTGCATGACTCACTGAATACGATCGCCAAGCCGTTC<br>CTGCGCGAATTCCTTGCTGAAAGTCCGGTCACCTGCGTAATCGCAGATGGCA<br>TTCTTTCGATGGCCGCGGACGTGGCAGAAGAGATTAATCTGCCGATTATTTA<br>CTTTCGTACGATTAGCGCATGCGCATTTTGGTCATATTTCAGTATTCCCGAAC<br>TGCTGCAGGCGGCAGAGCTGCCGCTGAAGGAGAATGGCATGGATATCACCC<br>TGACTAAAGTTAAGGGAATGGAAGATTTTCTCCGCGGACGTGACTTACCGTC<br>GTTCTGTCGTGTAAGCGATCTGACCAGTGCCGACTTTCGCTTGCTGTCCAGC<br>GAAACACGTCAGACGCCGCGCGCCCGCGGCTTAATTTTAAATACCTTCGAGG<br>ATTTGGAAGGACCTATCTTGAGTCAGATCCGTACCGTATGCCCGAACGTATAT<br>ACCATCGGCCCTGTGCACGCGCACCTCAAAACCCGCCTGGCTACCAAGTGC<br>ACTTCATCCAACTCCCTGTGGCAGGAAGATGAGAACTGTATTAACTGGCTTGA<br>TACCCACCCGCCGAAGTCGGTGTTGTACGTGTCGTTTGGTTCAATCGCAGGT<br>GTCACACGTGAAGAATTGCTGGAATTCTGGTATGGATTGGTCAACAGCGACC<br>AGAATTTTCTGTGGGTGATGCGTGCCGATCTTATCATCGGTCAGGAAGGCAA<br>GCACGAAATCTTAGAAGAGTTAGAACAGGGCACGAAAGCGCGTGGATACATG<br>GCCGACTGGGTACCTCAGGAGAAGGTTCTGGCGCACACTGCGATCGGCGGG<br>TTTCTGACGCATTGCGGCTGGAACAGCACCTTAGAATCCATTGTCGAGGGTG<br>TGCCGATGATCTGTTGGCCGCGCTTCGCCGACCAGCAGGTTAACTCTCGCTT<br>TATCGGGGAAGTATGGAAAATGGGCTTAGATATTAAGGACACCTGCGATCGT<br>GATATCATCGGTAAGAGTATCCGCGACCTTATGGAGAAACGCCGCGGGGAG<br>TTCTTACAGCGCACGGAACAAATGGCTAGCATGGCACGCCGTACCGTGAACG<br>AGGGCGGCTCTAGCTATATCAACCTGGATCGCCTGATTCAAGATATTCGCTTA<br>ATGTGCTTACCGCTGAAGCAATTTTCAGGTTCGAGAGACC
pEPQD0CM0560	CDS NbUGT76A4 (GenBank ID MT945330), codon optimised for *E. coli*	GGTCTCTAATGAAGATCCAGGAGCGTAAAATGAAGGTTGAGAAGCGCGAATC<br>AGTAGTGCTCGTACCTTACCCGTTTCAAGGTCACCTGACGCCGATGTTACAG<br>CTGGGAAGCATTCTCCACTCGCAGGGTTTCAGCGTGGTCGTAGCCCACACC<br>GAGTTTAACGCGCCTGACTACTCTAATCATCCGGAGTTTGTATTTCACTCGAT<br>GAATGATGGCCTGCAAGGCCGCGATATGTCGATGCCTAGCCTGGAGAACAT<br>GTACGACCTCAATGAAAACTGCAAAGCACCGCTGAAGGACTATCTGGCACGC<br>ATGATGGAAGATAACGGCGACGAGCTGGCCTGCATCGTGTACGATAATGTGA<br>TGTTCTTCGTCGACGACGTAGTAACACAGTTACGTATCCCTTCTATCGTGTTG<br>CGTACCTTTTCTACGACGTACTTACATTCAATGCTGACGATCTTGCAAAAGCC<br>GGATAAGTACCTTCCGTTCGAGGAAAGCCAATTATTAGACCCGCTCCCAGAA<br>CTGCACCCGTTGCGCAGCAAAGATATTCCGTTCCCGGTGATTGACAACACCG<br>TGCCGGAGCCTATCTTGGAGTTTTGCCGCGCCATGTCGGATATCGGGAGCTC<br>CGTGGCCACGATCTGGAATACCATGGAAGATCTTGAAAATAGTTTATTGCTGC<br>GTGTACAAGAGCACTATAAAGTACCGTTCTTCCCGATTGGCCCATTGCATAAG<br>ATGGCGCCGAGCACATCTTCGACGTCTTTGTTAGAGGAAGATGATTCTTGTAT<br>CGAATGGTTAGATAAGCAAGCACCGAACTCTGTGTTGTACGTGTCTTTAGGC<br>AGTCTTGTGAAAATTGACGATAAAGAACTCATCGAAACCGCGTGGGGCTTGG<br>CAAATTCTGAACAACCATTCCTGTGGGTGGTACGTCCGGGAAGCGTTTCGGG<br>ATTCCAGTGGACGGAAGCGCTGCCGGAAGGATTCGAAGAGACTATTGGTGA<br>GCGCGGCCGCATCGTAAAGTGGGCGCCCCAGAAGCAAGTACTGGCCCACCC<br>TGCAGTGGGTGGTTTCTTTACCCACTGCGGCTGGAACAGCACCTTGGAGTCG<br>ATCTGCGAGGAAGTTCCGGTTATTTGTCGTCCGATCCTCGCCGATCAGCCGG<br>TAAATGCGCGCTACCTGTCACAGATTTACATGGTGGGTTTAGAGTTAGAAGC<br>GACCGAGCGCTCAGTGATTGAAAAGACGGTTCGCAAGCTGATGCTCAGCGA<br>GGAAGGCAAGGACGTTAAGAAGCGTGTGGTTGAAATGAAGCAGAAAATTGTA<br>GCGGGCATGCAAATTGACGGCAGTAGCCACAAGAATTTAAACGACCTGGTCA<br>ATTTTATCAGCAGCCTTCCATCACGTACACCCCCGATGCCAGCAGTGGGCGG<br>CATTTTGACGAGCAATTACATTCCTTCGAAAACCATTATTGAATCGGGTTCGA<br>GAGACC
pEPQD0CM0561	CDS NbUGT85A104 (GenBank ID MT945331),	GGTCTCTAATGGGTACCTTGAATCAAACCGAACAGCCGATGAGTAAACCGCA<br>CGCCGTTTGTATCCCCTTCCCGGCCCAGGGTCACATTAACCCAATGTTGAAG<br>CTGGCAAAGCTGCTGCACATTCGCGGTTTCCACATTACGTTTGTGAATACGG<br>ATTTTAATCATCGTCGCTTACTGAAGTCTCGCGGCCCGAACGCGCTGAGTGG<br>TCTGCCGAGTTTCCGCTTTGAATCGATTCCGGACGGCTTACCGCCGTCGAAT<br>GACGATGCCACTCAGGACGTCCCGTCACTGTGCGAATCTTGCACCAAACTGT

	codon optimised for *E. coli*	GCCTTGCGCCCTTCCGTGAGTTGGTTACTCGTTTGAACAACAGTCTGAACTTC CCGCCAGTGACGTGTATCGTAAGTGACGCGGGCATGTCTTTTACACACGAGG TGTCCGAAGAGTTAGGCATCCCGAACGTCGCGTTCTGGACGGCGAGCGGCT GCGCGCTTTGGGCATTTCTGCAGTACCCGAAGCTCGTCGAGGAAGGCTACT GCCCGGTCAAGGACCACTCATATCTGACTAATGGTCACCTTGATACGATCATT GACTGGATCCCGGGGATGGAAGGGATTCGCTTGAAGAACCTTCCCTCATTTA TTCGCTCAACCGTAGATGAGCCGTCTTACATGGTTATTAAGTTCATCATGGAA GAGATTCTGGATAAGATCCCGAAGGCATCGGCGTTGATCCTGAATACCTTTG ACGCATTGGAAACCGACGTCCTTAAACCGATCTTAACGCTTTTCCCGACAGT GTACACCCTGGGCCCGTTCCATACAAGCCTGAACAACCAGACCCAGGATGA GGACCTTAAGAGCATCGGGAGCAACTTGTGGAAAGAGGACACACATTGCCTT GAGTGGCTTAATACAAAGAAGCCTAACTCTGTTGTTTACGTCAACTTCGGCTC GATCACCGTTCTGAGTCCTAAGCAACTTGTGGAGTTCGCTTGGGGTTTAGCA AACTCGAAGCTGAACTTTCTGTGGATCATCCGCTCCGACATCGTAAAAGGCG ACAGTCTGATTCTCCCGCCGGAGCTGCTTGCTGAAATCAAAGAGCGCGGACT CTTGTGCGGCTGGTGTCCACAGGAGCACGTCCTGTGCCATCCGAGCGTGGG CGGCTTCTTGACGCATTGCGGCTGGAACAGCACGTTCGAGAGCATTAGTTTC GGCGTCCCGATGCTGTGCTGGCCGTTCTTCGCCGACCAGCAGACCAACTGC TGGTTCATCTGCAACTGTCTTGGAGTGGGTATGGAGATCGACTCTCACGTTA AACGTGAGGTTATCGAGGAGTTGGTGAAGGAACTCATGATCGGGGAGAAGG GAATCGAACTGAAGGAGAACGCGCTCAAATGGAAACGTCTGACCGAGAAAAC CATTTCATCACCCGACGGCAGCTCGTACATGAACTTCGATAATCTGGTATCTC ACGTTCTTCTCCGTAAAGACTCATCCTTTCTCACTTGCGGTTCGAGAGACC
pEPYC0CM0470	CDS AtTGA1 (AT5G65210.2), contains a BbsI site	GGTCTCgAATGAATTCGACATCGACACATTTTGTGCCACCGAGAAGAGTTGGT ATATACGAACCTGTCCATCAATTCGGTATGTGGGGGGAGAGTTTCAAAAGCA ATATTAGCAATGGGACTATGAACACACCAAACCACATAATAATACCGAATAAT CAGAAACTAGACAACAACGTGTCAGAGGATACTTCCCATGGAACAGCAGGAA CTCCTCACATGTTCGATCAAGAAGCTTCAACGTCTAGACATCCCGATAAGATA CAAAGACGGCTTGCTCAAAACCGCGAGGCTGCTAGGAAAAGTCGCTTGCGC AAGAAGGCTTATGTTCAGCAACTGGAAACAAGCAGGTTGAAGCTAATTCAATT AGAGCAAGAACTCGATCGTGCTAGACAACAGGGATTCTATGTAGGAAACGGA ATAGATACTAATTCTCTCGGTTTTTCGGAAACCATGAATCCAGGGATTGCTGC ATTTGAAATGGAATATGGACATTGGGTTGAAGAACAGAACAGACAGATATGTG AACTAAGAACAGTTTTACACGGACACATTAACGATATCGAGCTTCGTTCGCTA GTCGAAAACGCCATGAAACATTACTTTGAGCTTTTCCGGATGAAATCGTCTGC TGCCAAAGCCGATGTCTTCTTCGTCATGTCAGGGATGTGGAGAACTTCAGCA GAACGATTCTTCTTATGGATTGGCGGATTTCGACCCTCCGATCTTCTCAAGGT TCTTTTGCCACATTTTGATGTCTTGACGGATCAACAACTTCTAGATGTATGCAA TCTAAAACAATCGTGTCAGCAAGCAGAAGACGCGTTGACTCAAGGTATGGAG AAGCTGCAACACACCCTTGCGGACTGCGTTGCAGCGGGACAACTCGGTGAA GGAAGTTACATTCCTCAGGTGAATTCTGCTATGGATAGATTAGAAGCTTTGGT CAGTTTCGTAAATCAGGCTGATCACTTGAGACATGAAACATTGCAACAAATGT ATCGGATATTGACAACGCGACAAGCGGCTCGAGGATTATTAGCTCTTGGTGA GTATTTTCAACGGCTTAGAGCCTTGAGCTCAAGTTGGGCAACTCGACATCGT GAACCAACGggTTCGTGAGACC
pEPYC0CM0471	CDS AtTGA2 (AT5G06950.1), domesticated to remove BsaI and BbsI sites	GGTCTCAAATGGCTGATACCAGTCCGAGAACTGATGTCTCAACAGATGACGA CACAGATCATCCTGATCTTGGGTCGGAGGGAGCACTAGTGAATACTGCTGCT TCTGATTCGAGTGACCGATCGAAGGGAAAGATGGATCAAAAGACTCTTCGTA GGCTTGCTCAAAACCGTGAGGCAGCAAGGAAAAGCAGATTGAGGAAGAAGG CTTATGTTCAGCAGCTAGAGAACAGCCGCTTGAAACTAACCCAGCTTGAGCA GGAGCTGCAAAGAGCAAGACAGCAGGGCGTATTCATTTCAGGCACAGGTGA CCAGGCCCATTCTACTGGTGGAAATGGTGCTTTGGCGTTTGATGCTGAACAT TCACGGTGGTTGGAAGAAAAGAACAAGCAAATGAACGAGCTGAGGTCTGCTC TGAATGCGCATGCAGGTGATTCTGAGCTTCGAATAATAGTCGATGGTGTGAT GGCTCACTATGAGGAGCTTTTCAGGATAAAGAGCAATGCAGCTAAGAATGAT GTCTTTCACTTGCTATCTGGCATGTGGAAAACACCAGCTGAGAGATGTTTCTT GTGGCTCGGTGGATTTCGTTCATCCGAACTTCTAAAGCTTCTGGCGAATCAG TTGGAGCCAATGACAGAGAGACAGTTGATGGGCATAAATAACCTGCAACAGA CATCGCAGCAGGCTGAAGATGCTTTGTCTCAAGGGATGGAGAGCTTACAACA GTCACTAGCTGATACTTTATCGAGCGGGACTCTTGGTTCAAGTTCATCAGGG AATGTCGCAAGCTACATGGGTCAGATGGCCATGGCAATGGGAAAGTTAGGTA CACTCGAAGGATTTATCCGCCAGGCTGATAATTTGAGACTACAAACATTGCAA CAGATGATAAGAGTATTAACAACGAGACAGTCAGCACGTGCTCTACTTGCAAT ACACGATTACTTCTCACGGCTACGAGCTCTAAGCTCCTTATGGCTTGCTCGAC CCAGAGAGggTTCGCGAGACC

pSD0KN04	CDS TaLUXA, codon optimised for *E. coli*<br><br>TRIAE_CS42_3AL_TGACv1_194142_AA0627410.1	GGTCTCTAATGGGGGAAGAAGCAGGCGGATATGGCTTTGATTTTGGCGGCG<br>GCGGCGGTGGATACGGTGGCTATGATGGCCGTGTGACTGAATGGGAAACCG<br>GGTTGCCTGGCTGTGATGAATTAACGCCTCTGTCCCAACCCTTGGTCCCGCC<br>TGGATTGGCGGCCGCCTTTCGTATTCCACCTGAACCCGGCCGTACGCTGTTG<br>GATGTACATCGTGCTAGTAGCGCGACGGTTAGCCGTCTGCGTAGCACATCAT<br>CCAGTCCTAGCTCAGGTAATGGGCATGCTGGAACTCATGCGAATGGTGGAAG<br>TTTTCCTTCGTTTCCGGGGAAAGGTGCCGCTGCTGGCGATGATTCTGGTAAT<br>CGTGATAATAATAGCGCAGAATCCGCGGGCGAGAAAGCTGCCGCAACAAAA<br>CGGGCACGTTTGGTCTGGACACCCCAACTCCATAAACGTTTTGTTGAAGTAG<br>TAGCTCATCTCGGAATTAAAAGTGCTGTTCCTAAAACGATTATGCAACTCATG<br>AATGTCGAAGGTTTAACGCGTGAAAATGTTGCGTCACATTTACAAAAGTATCG<br>TCTGTATGTAAAACGAATGCAAGGTCTGTCTAATGAAGGTCCTTCGGCGAGC<br>GATCATATTTTCGCCAGTACCCCTGTTCCCCCGTCGCTTCGTGAACCGCAAG<br>TTCCTCATGCTGCAGCAATGGCACCTGCGATGTATCATCATCATCCAGCACC<br>GATGGGCGGTGTAGCGGCAGGACATGGCGGCTATTATCAACAACAACATTCA<br>GGGCATGCTGTATATAACGGTTATGGTGGACATGGACATGGTGGCGGGGTTT<br>CATCATATCCACATTATCATCATGGAGATCAAGGTTCGGGAGACC
pSD0KN05	CDS TaLUXB, codon optimised for *E. coli*<br><br>TRIAE_CS42_3B_TGACv1_220755_AA0718150.1	GGTCTCTAATGGCGGGAACAACAGGTGGTTGGCGTAGCGGTCGTCGTGCAT<br>GTCGTGGAGCCACCAGTCTGCCTGGTTGTGATGAATTGACGCCCCTCTCTCA<br>ACCCCTCGTTCCGCCCGGTTTGGCGGCTGCCTTTCGTATTCCGCCAGAACCT<br>GGCCGGACCCTTCTTGATGTCCATCGTGCATCGTCTGCGACTGTTTCGCGTC<br>TGCGTTCGGCATCATCTAGTCCTTCAAGCGGGAATGGGCATGCGACCGGTG<br>GTGGTAGCTTTCCATCATTTCCGGGTAAAGCAGCCGCCGCTGCTGAAGCTGG<br>TGAAGATAGTGGTAATCGCGATAATAATTCAGCAGAAAGTGGTGGCGATAAA<br>AGTGCTGCTGCCGCAACCAAACGAGCTCGCCTCGTTTGGACTCCACAATTGC<br>ATAAACGTTTTGTCGAAGTTGTAGCCCATCTTGGCATTAAATCAGCAGTCCCG<br>AAAACGATTATGCAATTGATGAATGTAGAAGGTCTTACGCGTGAAAATGTGGC<br>AAGTCATCTGCAAAAGTATCGTCTGTATGTGAAACGTATGCAAGGTCTTAGCA<br>ATGAAGGCCCTAGCGCGTCAGATCATATTTTCGCATCAACTCCAGTACCGCC<br>TTCACTGCGTGAACCGCAAGTTCCAGTTCCCCATGCAGCTGCGATGGCACCA<br>GCAATGTATCATCATCATCCAGCTCCTATGGGTGGGGTTGCGGCAGGTCATG<br>GTGGTTATTATCAACAACAACATTCCGGTCATGCTGTGTATAACGGCTATGGT<br>GGCCATGGTCATGGTGGTGGTGTTTCGTCTTACCCACATTATCATCATGGAG<br>ATCAAGGTTCGGGAGACC
pSD0KN06	CDS TaLUXD, codon optimised for *E. coli*<br><br>TRIAE_CS42_3DL_TGACv1_250132_AA0862670.1	GGTCTCTAATGGGGGAAGAAGCAGGCGGTTATGATTTCGATTTTGGCGGCG<br>GCGGTGGCGGCTACGGTGGCTATGATGGCCGGGTTACCGAATGGGAAACTG<br>GTTTGCCGGGCTGTGATGAATTAACGCCTCTGAGTCAACCTCTCGTTCCGCC<br>CGGTTTAGCAGCTGCCTTTCGTATTCCACCCGAACCTGGTCGTACCCTGTTA<br>GATGTTCATCATGCATCAAGCGCGACGGTTAGCCGTCTGCGTTCAGCGAGCA<br>GCGGTAATGGGCATGCGGGAACTGGTGCAAATGGAGCGTCATTTCCTTCATT<br>TCCTGGTAAAGGTGCAGCTGCAGGCGACGATTCTGGTAATCGTGATAATAAT<br>AGCGCAGAATCTGCAGGTGAGAAAAGCGCTGCCGCCAAACGCGCACGATTG<br>GTCTGGACCCCACAATTGCATAAACGTTTTGTCGAAGTTGTCGCACATTTGGG<br>TATTAAATCAGCAGTTCCGAAAACGATTATGCAACTCATGAATGTTGAAGGTT<br>TAACACGTGAAAATGTTGCAAGTCATTTGCAGAAATATCGTTTGTATGTGAAA<br>CGTATGCAAGGTCTGAGTAATGAAGGTCCTTCTGCTAGTGATCATATCTTTGC<br>GTCAACCCCTGTTCCCCCATCCTTGCGTGAACCACAAGTTCCGCATGCTGCA<br>GCAATGGCGCCGGCTATGTATCATCATCATCCTGCACCTATGGGCGGCGTG<br>GCGGCGGGGCATGGCGGTTATTATCAACAACAACATAGTGGTCATGCAGTTT<br>ACAATGGATACGGTGGTCATGGTCATGGTGGCGGGGTATCATCGTATCCTCA<br>TTATCATCATGGAGATCAAGGTTCGGGAGACC
pHR0CM03	CDS TaLHYA, codon optimised for *E. coli*<br><br>TraesCS7A02G299400.2	GGTCTCAAATGGAAATCAACAGTAGCGGGGAAGAGACTGTCATTAAAGTCCG<br>TAAACCATATACCATTACCAAACAACGTGAACGTTGGACGGAAGCAGAACATA<br>AGCGCTTTCTGGAAGCGCTGAAGTTGTACGGTCGTGCATGGCAACGTATCGA<br>GGAACACGTGGGAACTAAAACCGCGGTACAGATTCGTTCGCACGCACAGAAA<br>TTCTTTACGAAACTGGAGAAAGAGGCCATTAACAACGGCACATCCCCAGGTC<br>AGGCACACGACATCGATATTCCGCCGCCTCGCCCGAAGCGCAAGCCTAATT<br>GCCCGTACCCCCGTAAGGGCTGCTTAAGCAGCGAAACGCCAACGCGCGAGG<br>TCCCTAAGAGCAGCGTTTCCCTGTCGAACAGTAACGCCGAGATGGCATCCAA<br>CGGTACCTTACAATTGACATGTATCCGCAAGCTGCAGCGTAAAGAACTGAGC<br>GAGAATGGGAGCTGTAGCGAGGTGATCAACATTTTCCGCGAGGCCCCGAGT<br>GCAAGCTTCAGCTCATCGAACAAATCTAGCAGTAACCACGGCGTGTCAGGCG<br>GCATCGAGCCTACCAAGACTGAGAATAAGGATATCGCGACTATGGAGCGTAA<br>GTCGACGAGTATCGACGTCGGCAAAGACGTCAAGGACATCAACGATCAAGA<br>GATGGAACGCAATAATCGTGTACATATTAGCTCAAACTACGATGGATCCCACG<br>AGGACTGCTTAGACAATTCCATGAAGCATATGCAGCTGAAACCCAACACCGT<br>TGAAACGACTTATACCGGGCAGCACGCCGCAAGCGCTCCCTTATATCAGATG

		AACAAAACGGGAGCCACGGGTGCACCGGATCCAGGGACGGAAGGCTCTCAC<br>CCAGACCAGACCTCTGACCGCGTTGGCGGCGCAAACGGCTCAATGGATTGT<br>ATTCACCCGACCCTGCCTGTAGACCCGAAGATCGGTTCATCTAGCACTGCGC<br>AAAGCTTCCCACATAATTACGCCGGGTTCGCCCCTACCATGCAGTGTCACTG<br>TAATCAGGACGCATATCGTTCGAGCCTGAACATGAGCAGTACATTTTCTAATA<br>TGCTGGTGTCTACTCTGCTCAGCAATCCTACCGTGCACGCTGCGGCGCGTCT<br>GGCCGCTAGCTATTGGCCGGCCGCAGATAGTAATATCCCAGTGGGCCCGAA<br>CCAGGAAGTGTTCGCCGAAAATGCCCAAGGCCGTCACATCGGAAGTCCGCC<br>CTCAATGGCGAGTGTAGTGGCGGCTACCGTGGCGGCAGCTTCAGCCTGGTG<br>GGCCACGCAAGGATTGCTTCCCCTGTTTGCGCCGCCTATGGCATTTCCCTTC<br>GTTCCTGTCCCGACCGCCTCATTCCCTACGACAGACGTGCAACGCGCCACC<br>GAAAATTGTCCCGTAGATAATGCGCCAAAAGAGTGTCAGGTCGCCCAAGAAC<br>AGGGACAGCCGGAAGCAATGATTGTGGTGGCAAGCAGCGGGAGTGATAAAA<br>GCGGTAAGGGCGAAGTTAGTCCGCATACAGAACTTAACATTAGCCCTGCGGA<br>CAAGGTAGAAACCACGCCCCCGACGGGTGCGGAAACCTCGGACGCCTTTGG<br>TAATAAGAAGAAACAAGACCGTAGTTCCTGCGGCTCGAATACCCCGAGCTCC<br>TCTGACGTCGAAGCCGAGCACGTGCCGGAAAATCAGGACCAGGCGAATGAT<br>AAAACCCAACAGGCTTGCTGTAGCAACAGCAGCGCAGGCGATATGAATCATC<br>GTCGCTTCCGCAATATCAGTTCTACGAACGACAGCTGGAAAGAGGTATCAGA<br>GGAAGGGCGCATGGCGTTTGACAAGTTGTTTTCCCGTGGTAAACTGCCACAG<br>TCCTTCTCACCGCCGCAGGCCGAGGGTTTAAAAGTTGTTCCGCGCGGCGAG<br>CAGGATGAGGCAACCACCGTAACCGTTGATCTGAATAAATCTGCAGCTGTAA<br>TGGATCACGAGCTGGACACCCTTGTAGGCCCGCGTGCCACGTTCCCGATCG<br>AGCTGAGCCATCTTAACATGAAAAGCCGTCGTACCGGTTTTAAGCCGTATAAA<br>CGCTGTAGTGTAGAGGCCAAAGAAAACCGCGTCCCTGCCGCAGATGAAGTC<br>GGCACGAAACGCATCCGTTTGGATTCGGAGCCGTCAACCGGTTCGTGAGAC<br>C
pHR0CM02	CDS TaNAMA1, codon optimised for *E. coli*<br><br>TraesCS6A02G 108300.2	GGTCTCAAATGCGTAGTATGGGTTCGAGTGATAGTTCCAGCGGGTCAGCCCA<br>GAAGGCGGCCCGTCACCAACACGAACCTCCTCCGCCACGTCAACGCGGGAG<br>CGCACCAGAATTGCCGCCTGGTTTTCGCTTTCATCCTACCGATGAAGAACTT<br>GTTGTCCATTATCTTAAGAAGAAAGCAGCGAAAGTTCCTCTTCCGGTAACGAT<br>TATTGCGGAAGTCGACCTGTATAAATTTGATCCCTGGGAACTGCCTGAAAAAG<br>CTACGTTTGGAGAACAAGAATGGTATTTCTTTTCTCCCCGTGATCGTAAATAT<br>CCGAATGGTGCCCGACCTAATCGCGCCGCAACCAGCGGTTATTGGAAAGCA<br>ACTGGTACTGATAAGCCGATTTTGGCGTCAGGTACTGGCTGTGGACTTGTAC<br>GTGAAAAGCTGGGTGTAAAGAAAGCCTTGGTCTTTTATCGTGGTAAACCACC<br>AAAAGGTTTAAAGACGAATTGGATTATGCATGAATATCGTTTAACGGATGCGA<br>GTGGTAGTACGACTACGTCGCGCCCGCCTCCTCCGGTCACGGGCGGTTCCC<br>GTGCAGCCGCGAGTCTGCGTGTTCGCACTCGCGTTGATCGTACCCTGGATG<br>ATTGGGTCCTTTGTCGTATTTATAAGAAGATTAATAAAGCAGCAGCCGGGGAC<br>CAACAACGGTCTACTGAATGTGAAGATTCAGTAGAAGATGCCGTAACTGCATA<br>TCCACTGTACGCAACTGCAGGTATGGCAGGCGCTGGAGCACACGGTTCTAAT<br>TATGCCTCGCCCTCTTTATTGCATCACCAAGATTCTCACTTTCTTGAAGGTTTG<br>TTTACGGCCGATGATGCGGGACTTTCTGCCGGGGCGACAAGCTTATCGCATC<br>TTGCAGCAGCAGCACGTGCCTCACCCGCGCCAACGAAGCAATTCCTGGCAC<br>CAAGCAGCTCGACACCCTTTAATTGGCTGGACGCATCTCCTGCAGGGATTCT<br>TCCTCAAGCCCGCAACTTTCCAGGATTCAATCGTTCGCGAAATGTGGGGAAC<br>ATGTCACTTAGCAGCACAGCGGATATGGCGGGTGCCGCAGGTAACGCTGTT<br>AATGCAATGTCAGCGTTCATGAACCCGCTGCCAGTACAGGATGGCACATATC<br>ACCAGCATCACGTGATCTTAGGTGCGCCTCTCGCCCCGGAAGCCACGACCG<br>GCGGAGCAACTTCCGGCTTCCAACACCCGGTTCAGGTGAGCGGAGTAAATT<br>GGAACCCTGGTTCGTGAGACC
pEPKK0CM0195	chrysanthemyl diphosphate synthase (CcCPPase) from *Chrysanthemum cinerariaefolium* P0C565.2, codon optimised for *E. coli*	GGTCTCTAATGACCACCACTTTATCGTCCAACCTCGATAGCCAGTTTATGCAG<br>GTCTACGAAACCCTTAAGTCAGAACTGATCCACGATCCTAGCTTCGAATTCGA<br>CGATGACAGCCGCCAATGGGTTGAACGCATGATCGATTATAACGTGCCAGGT<br>GGGAAAATGGTTCGCGGTTACAGCGTGGTCGATAGTTATCAGCTCTTGAAGG<br>GCGAGGAGCTGACAGAGGACGAGGCCTTCCTTGCATGCGCCTTAGGCTGGT<br>GTACCGAGTGGCTGCAGGCGTTCATTCTGGTGCTCGACGATATTATGGACGG<br>TAGCCATACTCGCCGTGGGCAGCCGTGCTGGTTCCGCCTTCCAGAAGTGGG<br>CGTGGTGGCGATCAACGACGGCGTACTCTTACGTAATCACGTTCACCGCATT<br>TTAAAGAAGTACTTTCAGGGCAAACCGTACTATGTTCATCTGCTCGATTTATTT<br>AACGAGACTGAGTTCCAGACTATTTCAGGCCAGATGATCGACACTATCTGCC<br>GCCTGGCGGGTCAGAAGGACTTGTCGAAATACACCATGACCTTAAATCGCCG<br>TATCGTGCAATATAAGGGCAGCTATTATTCCTGCTATCTGCCTATCGCATGCG<br>CGTTATTAATGTTCGGTGAAAACTTGGAAGATCACGTGCAGGTTAAGGATATT<br>CTGGTGGAGCTGGGCATGTACTACCAGATCCAAAACGACTACCTGGATACCT<br>TCGGCGACCCGGACGTGTTCGGTAAAACTGGTACGGACATCGAGGAGTGTA

		AATGCTCTTGGCTTATCGCTAAGGCCTTGGAGCTGGCAAATGAGGAGCAGAAGAAGATCCTGAGTGAGAATTACGGTATTAATGACCCTAGCAAAGTTGCGAAGGTTAAAGAGCTGTATCACGCGCTGGACCTTAAAGGAGCATACGAGGACTACGAAACCAACTTGTACGAAACCAGTATGACGAGTATCAAGGCACACCCCAATATCGCGGTTCAGGCAGTCCTCAAGAGCTGCTTAGAGAAAATGTACAAAGGCCACAAAGGTTCGAGAGACC
